# Phase diagrams of dune shape and orientation depending on sand availability

**DOI:** 10.1038/srep14677

**Published:** 2015-09-30

**Authors:** Xin Gao, Clément Narteau, Olivier Rozier, Sylvain Courrech du Pont

**Affiliations:** 1 Equipe de Dynamique des Fluides Géologiques, Institut de Physique du Globe de Paris, Sorbonne Paris Cité, Université Paris Diderot, UMR 7154 CNRS, 1 rue Jussieu, 75238 Paris Cedex 05, France; 2 Laboratoire Matière et Systèmes Complexes, Sorbonne Paris Cité, Université Paris Diderot, UMR 7057 CNRS, Bâtiment Condorcet, 10 rue Alice Domon et Léonie Duquet, 75205 Paris Cedex 13, France

## Abstract

New evidence indicates that sand availability does not only control dune type but also the underlying dune growth mechanism and the subsequent dune orientation. Here we numerically investigate the development of bedforms in bidirectional wind regimes for two different conditions of sand availability: an erodible sand bed or a localized sand source on a non-erodible ground. These two conditions of sand availability are associated with two independent dune growth mechanisms and, for both of them, we present the complete phase diagrams of dune shape and orientation. On an erodible sand bed, linear dunes are observed over the entire parameter space. Then, the divergence angle and the transport ratio between the two winds control dune orientation and dynamics. For a localized sand source, different dune morphologies are observed depending on the wind regime. There are systematic transitions in dune shape from barchans to linear dunes extending away from the localized sand source, and vice-versa. These transitions are captured fairly by a new dimensionless parameter, which compares the ability of winds to build the dune topography in the two modes of dune orientation.

Dunes have been primarily classified according to their shape considering both sand availability and wind directional variability^1^. The wind directional variability is usually measured by the RDP/DP dimensionless parameter. It is the ratio between the resultant drift potential (RDP, the norm of the mean sand flux vector) and the drift potential (DP, the mean of the norms of the individual sand flux vectors). In zones of low sand availability under nearly unidirectional winds (i.e., for high, close to one, RDP/DP-values), crescentic barchan dunes propagate on a non-erodible ground. Under the same wind conditions, transverse linear dunes form where sand availability increases. Star dunes are observed in major depositional centers exposed to wide multidirectional wind regimes (i.e., where the RDP/DP-value tends to zero). For moderate wind directional variability, linear dunes develop. Given the seasonal changes in wind direction on Earth, these linear dunes are widespread in terrestrial sand seas^2^. Dunes have also been classified according to their orientation. The crest lines of barchan dunes are approximately perpendicular to the resultant transport direction, whereas, by definition, the interlaced arms of star dunes have no unique orientation^3^. For linear dunes, *Hunter et al*.^4^ used the angle *ϕ* between their crestlines and the resultant sand transport direction to describe them as longitudinal (*ϕ* ≤ 15°), oblique (15 ° < *ϕ* < 75°) or transverse (*ϕ* ≥ 75°).

A significant breakthrough in understanding the relationships between bedform orientation and flow regime was the work of *Rubin and Hunter*^5^. When considering a bidirectional flow regime, they experimentally showed that sediment bedforms select the orientation for which the sum of the normal to crest components of the two transport vectors reaches its maximum value. Thus, they introduced the gross bedform-normal transport rule (from now the GBNR), which can be generalized to multidirectional flows^6^,^7^. Using this rule together with the dune type classification, a given wind regime should therefore be associated with a specific dune shape and a single orientation^8^,^9^. This single line of reasoning has been recently challenged on both experimental and field observations by *Courrech du Pont et al*.^10^. They have demonstrated that there are two modes for dune orientation depending on sand availability. Each mode is associated with a specific dune growth mechanism:

1. On an erodible sand bed, dunes grow in height and migrate selecting an orientation that maximizes the normal to crest components of transport, a result consistent with *Rubin and Hunter*^5^. This is the bed instability mode, which refers to the ability for a flat sand bed to organize in periodic bedforms as soon as there is sediment transport^11^.
2. On a non-erodible ground with a localized source of sediment, dunes grow by extension away from the source in the direction of the resultant sand flux at the crest. This is the fingering mode. Classical geomorphologic sand sources are depositional areas related to coastal or fluvial systems, pre-existing dunes or topographic obstacles.

Hence, the same wind regime can produce two dune orientations according to the sand availability. In addition, these two modes may locally coexist as a result of changes in sand availability or due to the development of superimposed bedforms.

*Courrech du Pont et al*.^10^ have developed a theoretical model to predict dune orientation as a function of a multidirectional wind regime and the prevailing growth mechanism. Model predictions have been confronted against a few experimental data and field examples. Although these comparisons support the validity of the approach, the model was not precisely checked against a wide range of wind conditions. Here we use numerical modeling to describe the diversity of dune patterns that can be produced under bidirectional wind regime using two different conditions of sand availability: an erodible sand bed or a non-erodible ground with a localized sand source. Over the entire parameter space of the bidirectional wind regime, the numerical results are compared to the prediction of the model of *Courrech du Pont et al*.^10^ using only one fitting parameter. This parameter is the wind speed-up, a key parameter in the physics of dunes, which introduces the effect of dune topography on the flow and on the subsequent sand flux over dune flanks. Furthermore, we study the phase diagram of linear dunes for the two dune growth mechanisms. Whereas linear dunes systematically occur in the bed instability mode, the dune morphology in the fingering mode exhibits transitions from barchans to linear dunes, and vice versa. We find that these changes in dune morphology cannot be associated to changes in the RDP/DP-value. Instead, these transitions can be captured by a dimensionless parameter that compares the dune height growth rates of the two growth mechanisms. Finally, we show a field example where this parameter map and the dune morphology map correspond.

## Results

**Figure 1:**
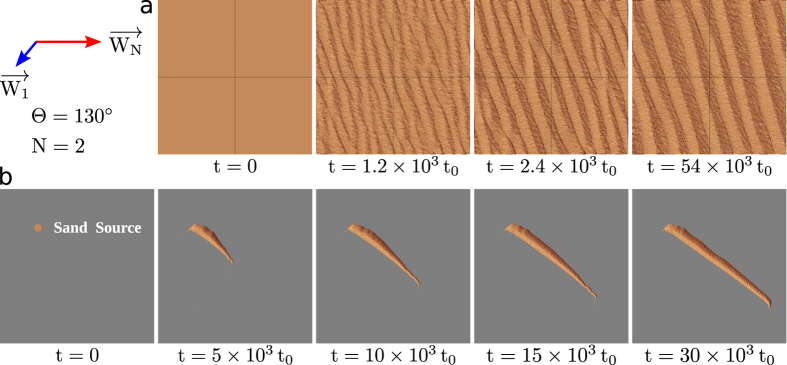
Formation and evolution of dune fields for *θ* = 130°, *N* = 2 and two conditions of sand availability. (**a**) Dunes grow in height and wavelength from a flat bed with no restriction in sand availability; (**b**) A finger dune extends on a non-erodible ground from a localized sand source. The red and blue arrows show the sand flux vectors of the dominant and secondary winds, respectively. The non-erodible ground is shown in gray. The cellular space has a square basis of side *L* = 600 *l*_0_. As shown by the orientation of the superimposed bedforms in (**a**) and the orientation of the finger tip in (**b**), images are taken after the secondary wind at the end of the cycle of wind reorientation.

In a cellular automaton dune model (see Methods), we set two types of numerical experiments to investigate the effect of sand availability. In a first set of experiments dedicated to conditions of high sand availability, a flat sand bed with a thickness larger than the flow depth ensures that bedforms never reach the non-erodible ground at the bottom of the cellular space. In addition, boundary conditions are periodic. The vertical edges of the cubic lattice are pasted together (see Fig. 1a at *t* = 0). In a second set of experiments dedicated to conditions of low sand availability, a circular source of sediment of diameter 20*l*_0_ is located upstream of a non-erodible ground (see Fig. 1b at *t* = 0). The input flux is not fixed, but there is always at least one layer of sedimentary cells on the circular source. Thus, the input flux adapts to the experimental conditions. In these simulations, boundary conditions are open to remove all sedimentary cells that reach the downstream border of the cellular space. For these two conditions of sand availability, we systematically investigate the dune morphology and orientation for periodic bidirectional wind regimes. The wind transport capacity is kept constant but the direction and the duration of the secondary wind are changed from one simulation to another (see Supplementary Note 1 and Table S1). Then, the periodic bidirectional wind regime is fully characterized by the divergence angle *θ* and the transport ratio *N* between the two winds. This transport ratio is simply the ratio between the time spent in the primary and secondary winds over a period of wind reorientation (i.e., *N* ≥ 1).

### Sand availability control on dune shape and orientation in bidirectional wind regimes

Figure 1a shows the development of bedforms starting from a flat sediment layer. Over short times, the bed instability mechanism is responsible for the development of dunes transverse to each individual wind (i.e., two distinct dune orientations may coexist). After a few cycles of wind reorientation, dunes are well established and exhibit a single alignment as they continue to grow in height by pattern coarsening (see Supplementary Note 2 and Movie S1). As shown in Fig. 1b, a completely different linear dune growth scenario occurs when sand availability is restricted to a localized source: a straight finger-like structure develops and elongates with a constant orientation. This finger extends but does not grow in height and width except at the source location. This shape is remarkably stable in space and time despite a permanent flux of sediment parallel to the crest on both sides of the dune (see Supplementary Note 2 and Movie S2). Taken together, Fig. 1a,b reveal the two dune growth mechanisms with obvious differences in orientation according to conditions of sand availability.

**Figure 2:**
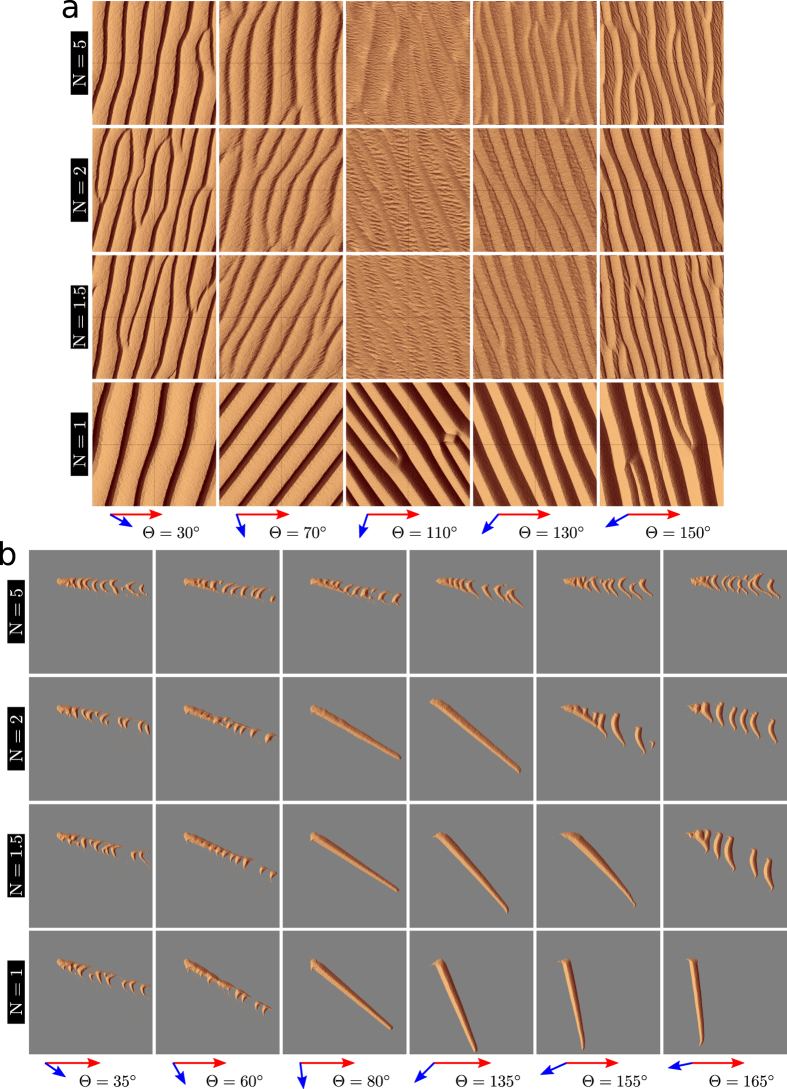
Steady-state dune field morphologies in the parameter space {*θ*, *N*} of bidirectional wind regimes for two conditions of sand availability. (**a**) Dunes grow in height and wavelength from a flat bed with no restriction in sand availability; (**b**) Dunes extend or propagate on a non-erodible ground from a localized sand source. The red and blue arrows show the sand flux vectors of the dominant and secondary winds, respectively. In all cases, the dominant wind blows from left to right. The non-erodible ground is shown in gray. The cellular space has a square basis of side *L* = 600 *l*_0_. As shown by the orientation of the superimposed bedforms in (**a**) and the orientation of the finger tip in (**b**), images are taken after the secondary wind at the end of the cycle of wind reorientation.

Figure 2a,b show the dune fields once they have reached a steady-state in morphology and orientation in the parameter space {*θ*, *N*} of bidirectional wind regimes under the same two conditions of sand availability as in Fig. 1. When bedforms grow from a flat sand bed (Fig. 2a), a periodic dune alignment is systematically observed. For *θ* < 90°, the two winds blow always from the same side of the dune and both participate to the formation of linear transverse dunes with gentle upstream slopes (~10°) and slip faces in the lee^12^. For *θ* > 90°, the two winds blow alternatively from both sides of the dune. As a consequence, the slip face switches alternatively from one side to the other. Dunes are more symmetric in shape with slopes of approximately 20°. Their orientations are always more perpendicular than parallel to the dominant wind direction. Hence, the dune aspect ratio seen by the secondary wind is smaller than the dune aspect ratio seen by the primary wind. It explains why superimposed bedforms perpendicular to the secondary wind are observed more easily than those perpendicular to the dominant wind (e.g., Fig. 2a for *θ* = 110°). However, they are transient dune features, which are rapidly blown away by the next wind (see Supplementary Note 2 and Movie S1). All these observed dune orientations are in good agreement with the GBNR.

When bedforms develop from a localized sand source, the steady-state dunes exhibit different shapes. Straight fingers are observed for a range of divergence angles, which depends on the transport ratio (e.g., 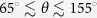 for *N* = 1.5). Outside of this range, finger-like structures extending away from a sand source are unstable and eventually break up into asymmetric barchans as already observed for {*θ* = 25°, *N* = 1} by *Reffet et al*.^13^ in underwater experiments. Trains of asymmetric barchans are always observed for *θ* < 60° or transport ratio *N* ≥ 3.5 (Fig. 2b; Supplementary Note 2 and Movie S3). For a given *N*-value smaller than 3.5, two transitions are observed when *θ* increases. For small *θ*-values, trains of barchans with small horns are observed (e.g., *θ* < 65° for *N* = 2). For intermediate *θ*-values, finger dunes are observed (e.g., 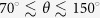 for *N* = 2). For large *θ*-values (e.g., *θ* > 150° for *N* = 2), asymmetric barchans are observed (see also *Taniguchi et al*.^14^ and *Parteli et al*.^15^). Unlike finger dunes, which are straight and seem to extend indefinitely, these asymmetric barchans have a curved crest line and arms with a limited extension. Their shape can be understood as the result of the interaction of the two growth mechanisms. The barchanoid base is transverse and stretched by two asymmetric arms. The arm that is upwind of the secondary flow has a smaller extension than the other one, whose tip tends to align in the mean transport direction (e.g., *θ* = 155°, *N* = 2 in Fig. 2b). Only one arm can elongate due to its position relative to the barchanoid base, which is a mobile source of sediment. In the numerical simulations with a localized sand source, the transition from a finger dune to asymmetric barchans occurs for a *θ*-value that depends on the transport ratio *N*, but it always larger than 90°. This is consistent with the flume experiments of *Taniguchi et al*.^14^ and the numerical simulations of *Parteli et al*.^15^ who studied the phase diagram of asymmetric barchans. The migration of asymmetric barchans modifies the orientation of the curvy elongated arm. However, in the entire parameter space {*θ*, *N*}, a clear orientation is given by either the finger trend, the alignment of the train of barchans or the elongated horn of asymmetric barchans (see Supplementary Note 3). Contrary to dunes that develop from an erodible ground (Fig. 2a), this orientation is always more parallel than perpendicular to the dominant wind direction.

Figure 2 as a whole shows the diversity of dune patterns and orientations that can be produced under two different conditions of sand availability. In the bed instability mode, note the abrupt change in dune orientation when the *θ*-value crosses 90°. Such a change does not occur for linear dunes in the fingering mode because finger dunes are longitudinal bedforms built by two winds blowing on either side of their crest whatever the divergence angle *θ*. Then, finger dunes can be sustained for *θ*-values smaller than 90° (see Supplementary Note 2 and Movie S4).

### Dune orientation

**Figure 3:**
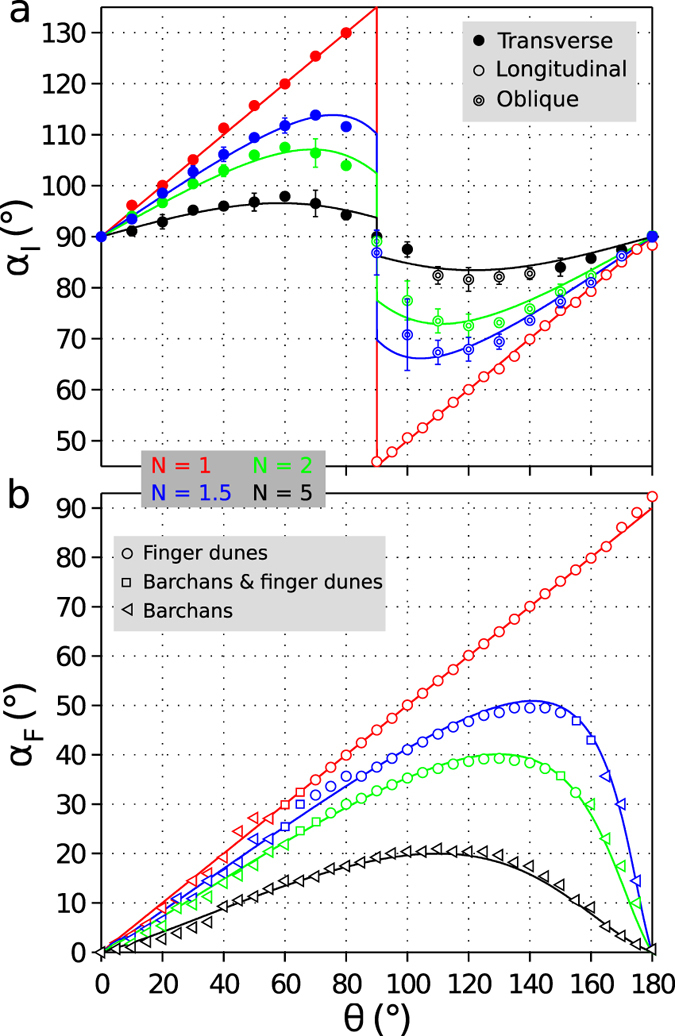
Dune field orientation with respect to the angle *θ* between the two winds for different transport ratio *N* and two conditions of sand availability. red *N* = 1, blue *N* = 1.5, green *N* = 2, black *N* = 5. (**a**) Dunes grow in height and wavelength from a flat bed with no restriction in sand availability; (**b**) Dunes extend or propagate on a non-erodible ground from a localized sand source. All orientations are defined with respect to the dominant wind direction using 2D spatial autocorrelation. Symbols differentiate finger dunes (circles), trains of barchan dunes (triangles) and coexistence of barchans and finger dunes (squares). For linear dunes, symbols also differentiate between transverse (full circles), oblique (double circles) and longitudinal dunes (open circles). Solid lines show the predicted dune orientation derived from equations (1) and (2) using *γ* = 1.6, the best-fit flux-up ratio. Error bars show the standard deviation for 10 realizations with different diffusion rates to evaluate the role of defects (see Supplementary Note 4). Note the good agreement between the prediction of the numerical model and the analytical solutions.

Using the numerical model output, we now turn to quantitative estimates of dune trends and sediment transport properties in the parameter space {*θ*, *N*} of bidirectional wind regimes. Symbols in Fig. 3a show the dune orientation *α*_*I*_ in the bed instability mode when dunes grow in height from the underlying sediment layer. For the same {*θ*, *N*} -values, symbols in Fig. 3b show the dune trend *α*_*F*_ in the fingering mode when dunes grow by extension away from a localized sand source. These two dune trends *α*_*I*_ and *α*_*F*_ measured in the numerical experiments compare well to the solutions of the theoretical model proposed by *Courrech du Pont et al*.^10^.

Let us consider a linear dune of height *H*, width *W* and orientation *α* with respect to 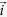 in the standard basis 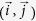. On the upstream slope of dunes, the convergence of flow lines results in an acceleration of flow speed and an increase of the bed shear stress^16^. Following *Courrech du Pont et al*.^10^ (see Supplementary Note 5), the sand flux at the crest is


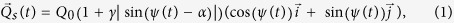


where *Q*_0_ is the saturated sand flux over a flat sand bed, *ψ* the wind direction and *γ* = *βH*/*W* the flux-up ratio. *β* is a dimensionless coefficient, which accounts for all the physical ingredients, e.g., roughness, that may affect the speed-up in addition to the apparent dune aspect ratio^17^,^18^,^19^. In our numerical simulations of dunes exposed to bidirectional wind regimes, only the time duration changes between the two winds, which ensures that *Q*_0_ is constant. For dunes that are large enough to integrate the entire cycle Δ*T* of wind reorientation, the dune height growth rate *σ* is assumed to scale like the characteristic flux divergence averaged over a cycle:


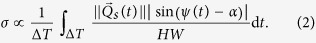


As in the GBNR^5^, the selected orientation *α*_*I*_ for the bed instability mode is the one with the largest growth rate *σ*_*I*_, such that d*σ*/d*α* = 0. For the fingering mode, the selected orientation *α*_*F*_ is the direction of the resultant sand flux at the crest of the dune, i.e., the orientation for which the normal to crest components of transport cancel each other. Obviously, these two orientations depend on the *γ*-value. In bidirectional wind regimes, *Courrech du Pont et al*.^10^ determined the ranges of dune trend that may be observed from *γ* = 0 to *γ* → +∞.

Inversely, given some specific dune orientations, we can estimate the *γ*-value that fits the best our data. We obtain *γ* = 1.6 using a least-squares method fitting simultaneously all the dune alignments measured in the numerical simulations. As shown in Fig. 3, both numerical and analytical results exhibit the same behavior across the entire parameter space {*θ*, *N*}. Such a good agreement supports that the two proposed dune growth mechanisms are at work in the numerical model, and that equations (1) and (2) capture the essential features of sediment transport at the crest of the simulated dunes. A flux-up ratio of 1.6 is consistent with the average values documented in experiments and aeolian dune fields^19^,^20^,^21^,^22^,^23^,^24^,^25^. This flux-up ratio corresponds to a fractional speed-up ratio smaller than 0.6 when considering a reasonable range of wind speed at a height of 10 m (e.g., *u*_10_ < 25 m s^−1^) and classical aeolian transport laws with a threshold wind velocity of 4.4 m s^−1^ (see Supplementary Notes 6 and 7).

### Phase diagram of finger linear dunes

**Figure 4:**
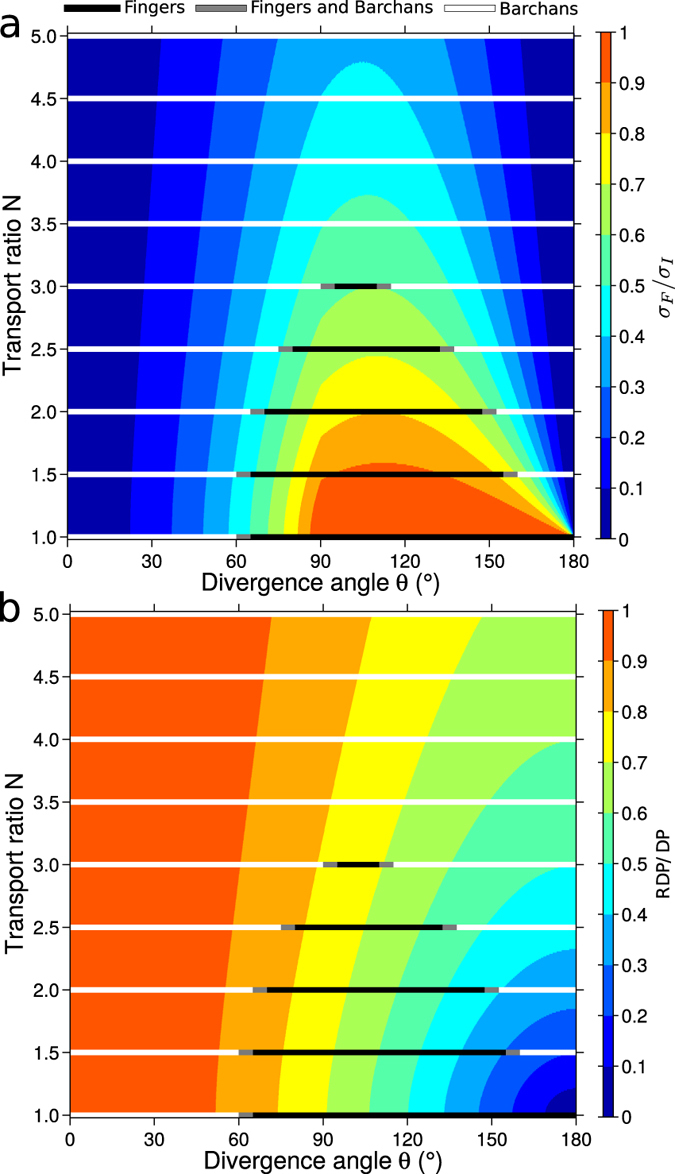
Transitions in dune shape in the parameter space {*θ*, *N*} when the dune develops from a localized sand source. (**a**) Growth rate ratio *σ*_*F*_/*σ*_*I*_ and (**b**) RDP/DP-value in the parameter space {*θ*, *N*} of bidirectional wind regimes. The growth rates {*σ*_*I*_, *σ*_*F*_} are computed with equation (2) using the corresponding dune orientations {*α*_*I*_, *α*_*F*_}, *γ* = 1.6 and the same {*H*, *W*}-values (i.e., the same dune shape). The *σ*_*I*_/*σ*_*F*_-value depends only on the wind regime. For numerical simulations with low sand availability and localized sand source (Fig. 2b), black and white lines show the zones of the parameter space {*θ*, *N*} in which fingers and barchans are observed, respectively. Gray lines indicate the zones for transition in dune shape.

We now address the transition from finger dunes to trains of barchans in the numerical experiments (Fig. 2b). For different transport ratios *N*, the black lines in Fig. 4 show the ranges of *θ*-values over which finger dunes are observed. This range decreases with an increasing transport ratio. Close to the transitions, it is common to observe fingers with a finite extension that emit barchans from their tip (e.g., for {60°, 1.5} or {155°, 2} in Fig. 2b). This shows that the two dune growth mechanisms coexist; superimposed dunes in the bed instability mode can develop on the top of dunes in the fingering mode to ultimately break them up into sets of barchans. Note that without a localized sand supply but starting from an initial sand pile, the source of sediment that potentially feeds the extension of a finger dune becomes mobile. In this case, a wider variety of dune shapes would be observed across the entire parameter space {*θ*, *N*}. This variety includes barchans, asymmetric barchans with an elongated arm, tear drop and chestnut-like dunes^13^,^14^,^15^.

Although a finger dune is longitudinal and aligned with the mean sand flux direction, its slopes are built and sustained by the two winds blowing from either side. Equivalently to the height growth rate *σ*_*I*_, one can derive the height growth rate *σ*_*F*_, which is the typical rate to shape a cross-section of a finger dune. In practice, the *σ*_*F*_-value is computed by injecting *α* = *α*_*F*_ in equation (2). Then, when the dune develops from a localized sand source, the relative contribution of the two dune growth mechanisms to the final dune shape can be quantified by the ratio *σ*_*F*_/*σ*_*I*_ between the height growth rates of the fingering and the bed instability modes. By definition *α*_*I*_ is the orientation for which the growth rate is maximum so that 0 < *σ*_*F*_/*σ*_*I*_ < 1.

**Figure 5:**
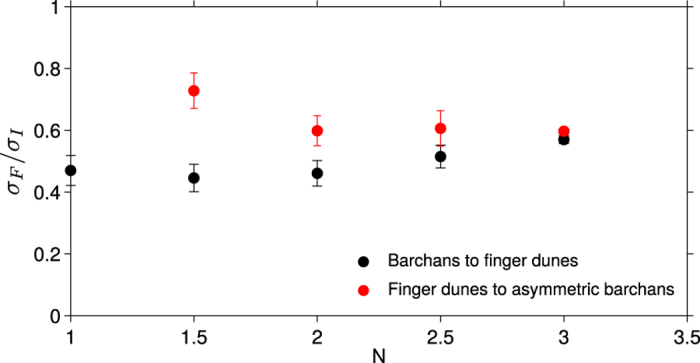
The *σ*_*F*_/*σ*_*I*_-values for transitions in dune shape as a function of the transport ratio *N*. For a given *N*-value and an increasing *θ*-value, dune morphology changes from barchans to finger dune (black symbols) and from finger dune to asymmetric barchans (red symbols). For a given *N*-value, error bars give the range of *σ*_*F*_/*σ*_*I*_-values over which the transition is observed.

Within the parameter space {*θ*, *N*}, Fig. 4a shows that transitions in dune shape are captured fairly well by the ratio *σ*_*F*_/*σ*_*I*_. Finger dunes are observed when 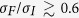. For a given *N*-value and an increasing *θ*-value, the transition from barchan to finger dunes occurs for a *σ*_*F*_/*σ*_*I*_-value which slightly increases with *N*, from 0.46 ± 0.03 when *N* = 1 to 0.57 ± 0.02 when *N* = 3 (see black dots in Fig. 5). For the same transport ratio *N* but larger angles of divergence *θ*, the transition from a finger dune to asymmetric barchans occurs for a *σ*_*F*_/*σ*_*I*_-value which slightly decreases with *N*, from 0.73 ± 0.05 when *N* = 1.5 to 0.6 ± 0.01 when *N* = 3 (see red dots in Fig. 5). For *N* ≥ 3.5, no stable finger dunes are observed. The *σ*_*F*_/*σ*_*I*_-value is then always smaller than 0.53. The parameter *σ*_*F*_/*σ*_*I*_ is also consistent with the finger domain extending up to 180° when *N* = 1. Note also that for a given *N*-value close to 1 and *γ* = 1.6, the *σ*_*F*_/*σ*_*I*_-value is maximum for divergence angles *θ* which are significantly larger than 90°. For larger *N*-values, this *θ*-value for which the *σ*_*F*_/*σ*_*I*_-value is maximum asymptotically tends to 90°. This is a consequence of the speed-up, the *σ*_*F*_/*σ*_*I*_-value is maximum for *θ* = 90° when *γ* = 0.

Despite the transition does not correspond to a sharp constant *σ*_*F*_/*σ*_*I*_-value (*σ*_*F*_/*σ*_*I*_ ≃ 0.60 ± 0.15, Fig. 5), this simple parameter quite separates the finger dune and barchan domains. The ratio 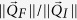 between the norms of sand fluxes associated to each mode, which also makes sense, captures as well the transition. Yet it is highly dependent of the *γ*-value (see Supplementary Note 5). It is worth mentioning that *σ*_*F*_/*σ*_*I*_ does not consider that the two modes may interact and is computed with a constant flux-up ratio (*γ* = 1.6) with no assumption on dune shape. It depends on the sole wind regime as the widely used RDP/DP parameter.

As the *σ*_*F*_/*σ*_*I*_-value, the RDP/DP-value depends on the divergence angle *θ* and the transport ratio *N* between the two winds. But this classic measurement of the wind variability fails to capture all the transitions in dune shape (Fig. 4b). Although the transitions from barchan to finger dunes are observed for 0.7 < RDP/DP < 0.9, the transitions from finger to asymmetric barchans correspond to a wide range of RDP/DP-values, from 0.6 for *N* = 3 to 0 for *N* → 1. Basically, the RDP/DP cannot distinguish dune shape in complex wind regime if different sand flux components cancel each other.

Hence, we infer that, in zone of low sand availability, *σ*_*F*_/*σ*_*I*_ can be used to predict the dune field morphology. For large *σ*_*F*_/*σ*_*I*_-values, the fingering mode should prevail and linear dunes aligned parallel to the resultant sand flux should be observed. For small *σ*_*F*_/*σ*_*I*_-values, finger dunes are unstable, the bed instability should prevail and migrating barchans should be observed.

## Discussion

To test these predictions against natural conditions in modern terrestrial sand seas, we use field images supplied by Google Earth and wind data supplied by the ERA-Interim dataset^26^,^27^. This global atmospheric reanalysis provides updated wind speed and orientation at 10 m above ground since 1979, with a 0.25° horizontal resolution and a 6 h time resolution. Undisturbed saturated sand fluxes *Q*_0_ are computed with the transport law of *Ungar and Haff*^28^, which scales as the wind velocity square minus the transport onset velocity square. The transport onset friction velocity is chosen to be 19 cm s^−1^, consistently with the measurements of *Iversen and Rasmussen*^29^. {*α*_*I*_, *α*_*F*_} and growth rates {*σ*_*I*_, *σ*_*F*_} are then computed using *γ* = 1.6 (see Supplementary Note 6).

**Figure 6:**
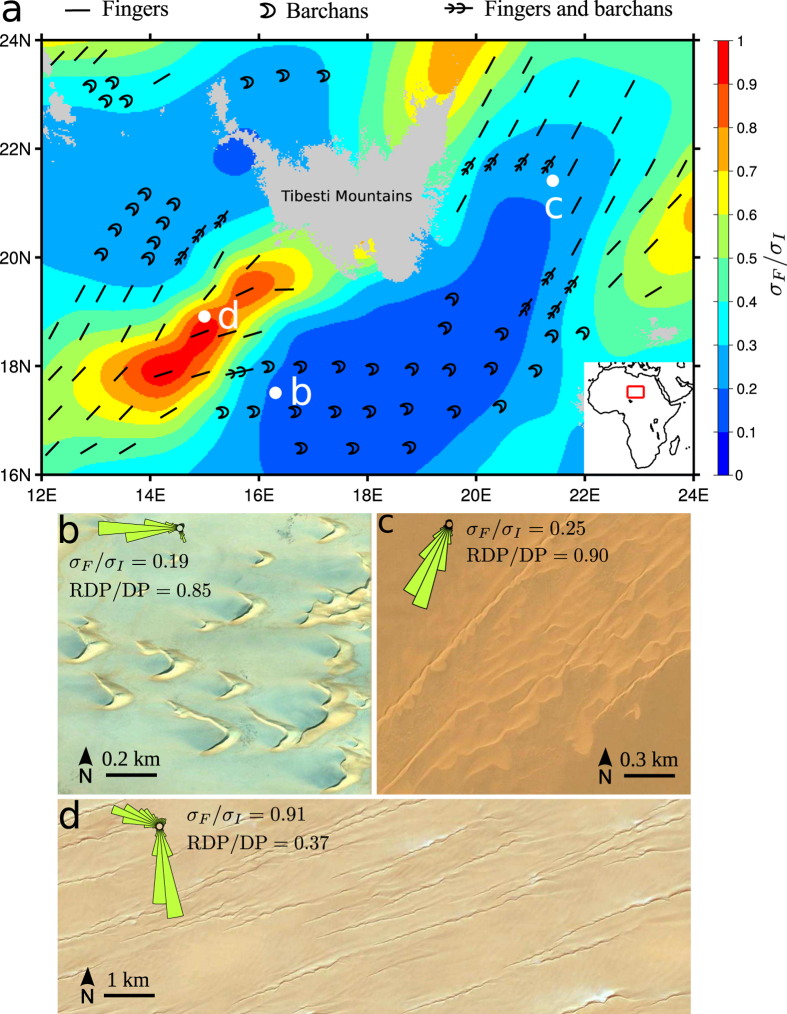
Transitions in dune shape around the Tibesti Massif (east central Sahara), a zone of low sand availability. (**a**) Map of *σ*_*F*_/*σ*_*I*_-value derived from the wind data. The continuous map is generated by linear interpolation based on the horizontal spatial resolution of 0.25° × 0.25° of the wind data. Local dune fields exhibit (**b**) barchans (image credit: Google Earth), (**c**) fingers breaking up into barchans (image credit: Google Earth) and (**d**) fingers elongating in the direction of the resultant sand flux (image credit: Google Earth). All these different dune types are reported in (**a**) using black symbols. Insets in (**b**–**d**) show the local flux roses. The map used in figure (**a**) is generated by GMT (The general mapping tools).

Here, we concentrate on the ergs around the Tibesti Massif in east central Sahara, where *Wilson*^30^ and *Mainguet and Callot*^31^ have identified major changes in flow orientations and a variety of dune shapes. In this area of low sand availability where the inter-dune area is free of sand or composed of coarse-grain sediment, we identify and locate the different dune types from recent satellite images. Then, we compare the spatial distribution of barchans and linear finger dunes to the *σ*_*F*_/*σ*_*I*_-value map derived from the wind data (Fig. 6a). Barchans occur in zones where *σ*_*F*_/*σ*_*I*_ < 0.2 (Fig. 6b), whereas linear finger dunes are always observed where *σ*_*F*_/*σ*_*I*_ > 0.4 (Fig. 6d). In most cases, zones of intermediate values of *σ*_*F*_/*σ*_*I*_ exhibit both barchans and finger dunes (Fig. 6c). Although the transition is not observed for the same *σ*_*F*_/*σ*_*I*_-value in the simulations and in the field, these observations are consistent with our numerical results and predictions. The quantitative discrepancy between the transition values of *σ*_*F*_/*σ*_*I*_ could be ascribed to non fully accurate ERA-Interim wind data in this mountainous area, which is distant of any meteorological station.

**Figure 7:**
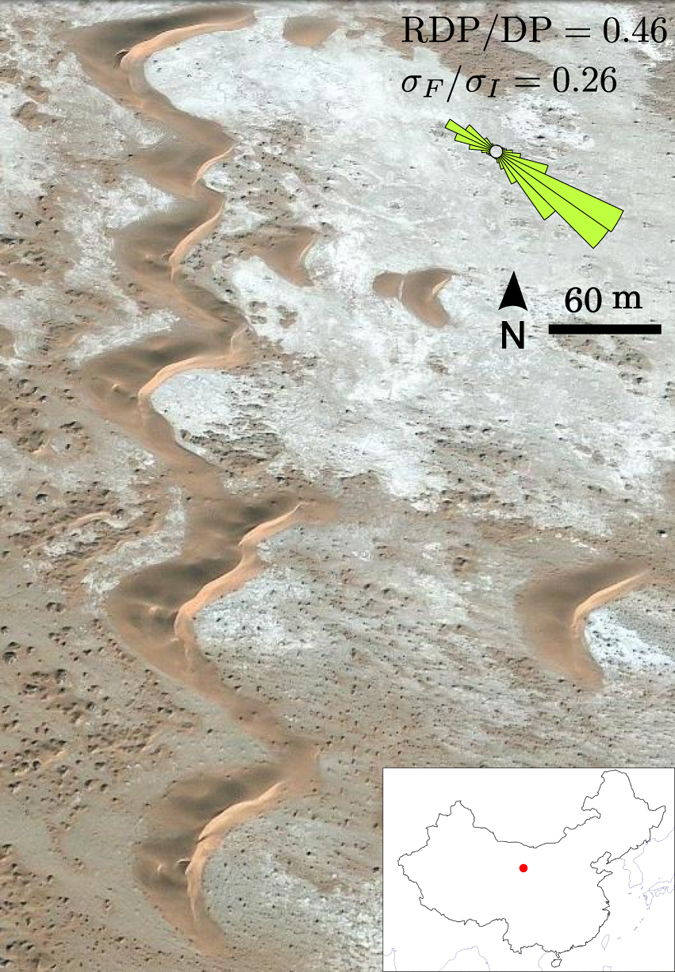
Barchan dunes in a zone of low RDP/DP and *σ*_*F*_/*σ*_*I*_-values. These dunes are located in a sand corridor between the Badain Jaran and the Tengger deserts (China, 39°23′25′′ N, 102°59′26′′ E, image credit: Google Earth). Inset shows the local sand flux roses. In this area, the mean RDP/DP-value and the flux roses derived from the ERA-Interim data are in agreement with the direct measurements of *Zhang et al*.^38^, ten kilometers away from the dunes. The map used in this figure is generated by GMT (The general mapping tools).

In the ergs around the Tibesti Massif where linear dunes occur, dunes always roughly align with the resultant sand flux. It indicates that these linear dunes recognized as seifs by *Bagnold*^32^ or silks by *Mainguet and Callot*^31^ are finger dunes that grow by extension from localized depositional areas. Note that zones of transition in this field coincide to places where the wind tends to be more unidirectional. It corresponds to the transition from barchans to finger dunes when the *θ*-value increases for a given *N*-value, which is also captured by the RDP/DP parameter (Fig. 4b). Nevertheless, barchan dunes are not restricted to large RDP/DP values. In agreement with our predictions, barchans can occur in zones of moderate RDP/DP-value, providing that *σ*_*F*_/*σ*_*I*_ is small enough. This is for example the case in sand corridors between the Badain Jaran and the Tengger deserts where two wind components cancel each other (see Fig. 7).

Our numerical experiments show that sand availability acts on both dune shape and orientation. For a given condition of sand availability, the dune shape and orientation are uniquely determined by the wind regime. For periodic bidirectional wind regimes and two different conditions of sand availability, we document all the possible dune morphologies and orientations in the numerical model. In quantitative agreement with the model of *Courrech du Pont et al*.^10^, these orientations can be derived from the sand flux at the crest and two independent dune growth mechanisms. When dunes develop from an erodible sand bed, linear dunes are observed. Their orientation maximizes the dune height growth rate in agreement with the GBNR^5^. Depending on the wind regime, their orientation can be transverse, oblique or longitudinal to the direction of the resultant transport direction. When dunes develop from a localized source of sediment, two major dune types are observed: finger linear dunes and barchans. Fingers grow by extension as a result of the sediment flux parallel to the dune crest while the dune topography is built and sustained by the sediment flux transverse to the dune crest. The orientation of finger linear dunes is the one of the sand flux resultant at the dune crest. For strongly asymmetric wind regime (*θ* → 0° or *N* > 5) or if transport components cancel each other (*θ* → 180°), fingers break up into trains of barchans because superimposed dunes in the bed instability mode develop and propagate parallel to the main dune crest. In zones of low sand availability, the dune morphology (i.e., barchan or finger dune) can be inferred from the growth rate ratio *σ*_*F*_/*σ*_*I*_, which captures fairly well the transitions in dune types both in the simulations and in a field example. Although the chosen field example supports the validity of the approach for a multidirectional wind regime, the robustness of the parameter *σ*_*F*_/*σ*_*I*_ to predict the dune morphology should be tested in complex wind regimes. The transition in dune shape would also deserve to be investigated further by exploring more precisely the dynamics of superimposed bedforms and the role of cohesion^33^,^34^. In addition, it would be interesting to systematically test the stability and the behavior of dune fields to a spatial or a temporal change of wind regime or sand supply.

## Methods

The numerical model combines a cellular automaton of sediment transport with a lattice-gas cellular automaton for high Reynolds-flow simulations^11^,^35^,^36^. This model is a fully coupled system in which there is a complete feedback mechanism between the flow and the evolving bed elevation profile.

In the cellular automaton of sediment transport, the lattice is made of cubic cells of elementary length scale *l*_0_. A cell is defined by its state: fluid, mobile and immobile sediment. Erosion, transport, deposition, diffusion and avalanching are simulated using different sets of transitions between pairs of nearest neighbor cells. Meanwhile, the particles of the lattice-gas cellular automaton can propagate and collide within the fluid state of the model of sediment transport. Local wind speed and direction are computed by averaging the velocity vectors of the fluid particles in space and time. There is a permanent effect of topography on the flow because the fluid particles rebound back along their initial trajectories when they impact on sedimentary cells. Reciprocally, the flow continuously affects the topography through sediment transport in zones where the basal shear stress is strong enough to mobilize sedimentary particles. In order to determine the effect of flow velocity on the erosion rate Λ_*e*_, we locally compute the spatial variation of the tangential velocity profile. In the referential of the topography, it writes


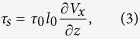


where *τ*_0_ is the stress scale of the model and *V*_*x*_ is dimensionless and expressed as a number of fluid particles. Then, we consider that the erosion rate Λ_*e*_ is linearly related to the bed shear stress *τ*_*s*_ beyond a threshold value *τ*_1_


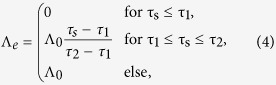


where Λ_0_ is a constant rate, *τ*_1_ is the threshold for motion inception and *τ*_2_ is a parameter to adjust the slope of the linear relationship. By definition, (*τ*_*s*_ − *τ*_1_) is the excess shear stress from which we can account for the feedback mechanism of the bed shear stress on topography. This erosion rate and the rates associated to each set of transition translate into the model the characteristic time scale of the underlying physical processes. The characteristic length and time scales {*l*_0_, *t*_0_} of the model are entirely defined with respect to the most unstable wavelength for the formation of dunes and the saturated sediment flux^11^. Therefore, the model can be used to quantitatively address dune morphodynamics in various physical environments (see Table 3 in *Zhang et al*.^37^).

The lattice gas cellular automaton and the model of sediment transport have two independent time scales. As in nature, the evolution of dune topography in the numerical simulations is many orders of magnitude slower than the evolution of the surrounding turbulent flow. Practically, the temporal coupling is controlled by the dynamic topography and we set the frequency *f*_*c*_ at which the propagation cycles of the flow model occur within the cellular space of the model of sediment transport. Thus, for high *f*_*c*_-values, expressed in units of frequency 1/*t*_0_, the flow always adapts to the current state of the topography. When the overall wind conditions are changing (e.g., after rotation), a stabilization of the flow is systematically performed before starting the processes of sediment transport.

A rotation of the cellular space simulates a change in wind orientation. After each rotation, the model of sediment transport is restarted once the flow has reached a steady-state. We analyze periodic bidirectional wind regimes with two winds {**W**_**N**_, **W**_**1**_} of equal strength but of different durations {Δ*T*_N_, Δ*T*_1_}. The saturated flux over a flat sand bed has a constant value *Q*_0_ over the entire period of wind reorientation Δ*T* = Δ*T*_N_ + Δ*T*_1_. The bidirectional wind regime is defined by the divergence angle *θ* ∈ [0; 180°] and the transport ratio *N* = Δ*T*_N_/Δ*T*_1_ between the two winds. **W**_**N**_ is the dominant wind so that *N* ≥ 1. More details are given in Supplementary Notes.

## Additional Information

**How to cite this article**: Gao, X. *et al*. Phase diagrams of dune shape and orientation depending on sand availability. *Sci. Rep*. **5**, 14677; doi: 10.1038/srep14677 (2015).

## Supplementary Material

Supplementary Information

Supplementary Movie S1

Supplementary Movie S2

Supplementary Movie S3

Supplementary Movie S4

## References

[b1] Wasson R. & Hyde R. Factors determining desert dune types. Nature 304, 337–339 (1983).

[b2] Pye K. & Tsoar H. Aeolian Sand and Sand Dunes (Unwin Hyman, London, 1990).

[b3] Zhang D., Narteau C., Rozier O. & Courrech du Pont S. Morphology and dynamics of star dunes from numerical modelling. Nat. Geosci. 5, 463–467 (2012).

[b4] Hunter R. E., Richmond B. M. & Alpha T. R. Storm-controlled oblique dunes of the Oregon coast. Geol. Soc. Am. Bull. 94, 1450–1465 (1983).

[b5] Rubin D. M. & Hunter R. E.. Bedform alignment in directionally varying flows. Science 237, 276–278 (1987). 17772055 10.1126/science.237.4812.276

[b6] Werner B. & Kocurek G. Bed-form dynamics: Does the tail wag the dog? Geology 25, 771–774 (1997).

[b7] Ping L., Narteau C., Dong Z., Zhang Z. & Courrech du Pont S. Emergence of oblique dunes in a landscape-scale experiment. Nat. Geosci. 7, 99–103 (2014).

[b8] Kocurek G. & Ewing R. Aeolian dune field self-organization-implications for the formation of simple versus complex dune-field patterns. Geomorphology 72, 94–105 (2005).

[b9] Fenton L. K., Michaels T. I. & Beyer R. A. Inverse maximum gross bedform-normal transport 1: How to determine a dune-constructing wind regime using only imagery. Icarus 230, 5–14 (2014).

[b10] Courrech du Pont S., Narteau C. & Gao X. Two modes for dune orientation. Geology 42, 743–746 (2014).

[b11] Narteau C., Zhang D., Rozier O. & Claudin P. Setting the length and time scales of a cellular automaton dune model from the analysis of superimposed bed forms. J. Geophys. Res. 114, F03006 (2009).

[b12] Zhang D., Narteau C. & Rozier O. Morphodynamics of barchan and transverse dunes using a cellular automaton model. J. Geophys. Res. 115, F03041 (2010).

[b13] Reffet E., Courrech du Pont S., Hersen P. & Douady S. Formation and stability of transverse and longitudinal sand dunes. Geology 38, 491–494 (2010).

[b14] Taniguchi K., Endo N. & Sekiguchi H. The effect of periodic changes in wind direction on the deformation and morphology of isolated sand dunes based on flume experiments and field data from the Western Sahara. Geomorphology 179, 286–299 (2012).

[b15] Parteli E. J. . Origins of barchan dune asymmetry: insights from numerical simulations. Aeolian Res. 12, 121–133 (2014).

[b16] Jackson P. & Hunt J. Turbulent wind flow over a low hill. Q. J. Roy. Meteorol. Soc. 101, 929–955 (1975).

[b17] Britter R., Hunt J. & Richards K. Air flow over a two-dimensional hill: Studies of velocity speed-up, roughness effects and turbulence. Q. J. Roy. Meteorol. Soc. 107, 91–110 (1981).

[b18] Fourrière A., Claudin P. & Andreotti B. Bedforms in a turbulent stream: formation of ripples by primary linear instability and of dunes by nonlinear pattern coarsening. J. Fluid Mech. 649, 287 (2010).

[b19] Courrech du Pont S. Dune morphodynamics. Comptes Rendus Physique 16, 118–138 (2015).

[b20] Lancaster N., Nickling W., Neuman C. & Wyatt V. Sediment flux and airflow on the stoss slope of a barchan dune. Geomorphology 17, 55–62 (1996).

[b21] Wiggs G. F., Livingstone I. & Warren A. The role of streamline curvature in sand dune dynamics: evidence from field and wind tunnel measurements. Geomorphology 17, 29–46 (1996).

[b22] Neuman C. M., Lancaster N. & Nickling W. Relations between dune morphology, air flow, and sediment flux on reversing dunes, Silver Peak, Nevada. Sedimentology 44, 1103–1111 (1997).

[b23] Walker I. J. & Nickling W. G. Simulation and measurement of surface shear stress over isolated and closely spaced transverse dunes in a wind tunnel. Earth Surf. Processes Landforms 28, 1111–1124 (2003).

[b24] Wu X., Zou X., Zheng Z. & Zhang C. Field measurement and scaled-down wind-tunnel model measurement of airflow field over a barchan dune. J. Arid. Environ. 75, 438–445 (2011).

[b25] Claudin P., Wiggs G. & Andreotti B. Field evidence for the upwind velocity shift at the crest of low dunes. Boundary-Layer Meteorol. 148, 195–206 (2013).

[b26] Uppala S. M. . The ERA-40 re-analysis. Q. J. Roy. Meteorol. Soc. 131, 2961–3012 (2005).

[b27] Dee D. . The ERA-interim reanalysis: Configuration and performance of the data assimilation system. Q. J. Roy. Meteorol. Soc. 137, 553–597 (2011).

[b28] Ungar J. & Haff P. Steady state saltation in air. Sedimentology 34, 289–299 (1987).

[b29] Iversen J. D. & Rasmussen K. R. The effect of wind speed and bed slope on sand transport. Sedimentology 46, 723–731 (1999).

[b30] Wilson I. G. Desert sandflow basins and a model for the development of ergs. Geogr. J. 137, 180–199 (1971).

[b31] Mainguet M. & Callot Y. L’Erg de Fachi-Bilma (Tchad-Niger): Contribution à la connaissance de la dynamique des ergs et des dunes des zones arides chaudes (Éd. du Centre national de la recherche scientifique, 1978).

[b32] Bagnold R. A. The Physics of Blown Sand and Desert Dunes (Chapman and Hall, London, 1941).

[b33] Rubin D. M. & Hesp P. Multiple origins of linear dunes on Earth and Titan. Nat. Geosci. 2, 653–658 (2009).

[b34] Araújo A. D., Parteli E. J., Pöschel T., Andrade J. S. & Herrmann H. J. Numerical modeling of the wind flow over a transverse dune. Sci. Rep. 3, 2858 (2013). 24091456 10.1038/srep02858 PMC3790208

[b35] Narteau C., Le Mouël J., Poirier J., Sepúlveda E. & Shnirman M. On a small-scale roughness of the core-mantle boundary. Earth Planet. Sci. Lett. 191, 49–60 (2001).

[b36] Rozier O. & Narteau C. A real-space cellular automaton laboratory. Earth Surf. Processes Landforms 39, 98–109 (2014).

[b37] Zhang D., Yang X., Rozier O. & Narteau C. Mean sediment residence time in barchan dunes. J. Geophys. Res. Earth Surf. 119, 451–463 (2014).

[b38] Zhang Z., Dong Z. & Li C. Wind regime and sand transport in China’s Badain Jaran Desert. Aeolian Res. 17, 1–13 (2015).

