# Supplementary material for: Phase diagrams of dune shape and orientation depending on sand availability
Source: Sci Rep. 2015 Sep 30;5:14677. doi: 10.1038/srep14677 (PMC4588573; doi:10.1038/srep14677)
Supplement: Supplementary Information [file srep14677-s1.pdf]

Phase diagrams of dune shape and orientation depending on sand availability
— Supplementary Information —

Xin Gao[1*], Clément Narteau[1], Olivier Rozier[1] and Sylvain Courrech du Pont[2]

[1]Equipe de Dynamique des Fluides Géologiques, Institut de Physique du Globe de Paris, Sorbonne Paris Cité, Univ Paris Diderot, UMR 7154 CNRS, 1 rue Jussieu, 75238 Paris Cedex 05, France.

[2]Laboratoire Matière et Système Complexes, Sorbonne Paris Cité, Univ Paris Diderot, UMR 7057 CNRS, Bâtiment Condorcet, 10 rue Alice Domon et Léonie Duquet, 75205 Paris Cedex 13, France.

Contents

1 Supplementary Note 1 **2**

2 Supplementary Note 2 **2**

3 Supplementary Note 3 **4**

4 Supplementary Note 4 **5**

5 Supplementary Note 5 **5**

6 Supplementary Note 6 **12**

7 Supplementary Note 7 **16**

*Correspondence to: gao@ipgp.fr

1 Supplementary Note 1: Wind regimes in the numerical simulations

For all the numerical simulations presented in the main manuscript, Table S1 shows the divergence angle θ and the transport ratio N between the two winds as well as the corresponding time durations ΔT_N and ΔT_1 of the dominant and secondary winds. The entire cycle of wind reorientation $\Delta T = \Delta T_N + \Delta T_1$ consists only of two changes in wind orientation.

When a dune field has reached a steady-state (see Figure 2 of the main manuscript), it keeps a constant orientation over the entire cycle of wind reorientation. This is because the duration of each wind is shorter than the turnover time of dunes. (see Supplementary Note 5 for details). Nevertheless, dune shape may slightly change during individual winds, mainly because of the development of superimposed bedforms perpendicular to the current wind orientation. Figure S1 shows two examples of the steady-state dune fields in the bed instability mode after the dominant and the secondary winds. In this mode, the alignment of the primary dunes is more parallel than perpendicular to the secondary wind. The apparent dune aspect ratio seen by this secondary wind is then smaller (i.e., gentle slope), promoting the development of superimposed bedforms.

In the main manuscript dune orientation is measured at the end of the dominant and secondary winds. All the images of dune fields are taken after the secondary wind $\mathbf{W_1}$ at the end of the cycle of wind reorientation.

2 Supplementary Note 2: Formation of dunes in bidirectional wind regimes from numerical modeling (4 Movies)

To illustrate the effect of sand availability on dune shape and orientation, Movies S1 to S4 show the formation of dunes in the numerical model using two different initial conditions and asymmetric bidirectional wind regimes.

Movie S1. To illustrate dune growth in the bed instability mode (i.e., high sand availability), Movie S1 shows the formation and evolution of bedforms from a flat sand bed exposed to a bidirectional wind regime with $\theta = 150°$ and $N = 2$ (see the steady-state dune fields associated with this dune growth mechanism in Figure 2a of the main manuscript). Dunes grow in height from the underlying sedimentary layer to finally form periodic ridges of oblique dunes (i.e., there is an angle of 55.3° between the dune trend and the resultant sand flux on a flat sand bed).

Movie S2. To illustrate dune growth in the fingering mode (i.e., low sand availability), Movie S2 shows the development of bedforms on a non-erodible ground from a localized sand source exposed to bidirectional wind regime with $\theta = 135°$ and $N = 2$ (see the steady-state dune fields associated with this dune growth mechanism in Figure 2b of the main manuscript). In this region of the parameter space $\{\theta, N\}$ of bidirectional wind regimes, finger dunes extend away from the sand source without moving sideways and with no change in orientation and shape. There is no lateral migration because, at the dune crest, the normal components of transport cancel each other. Dune extension is then driven by the permanent sediment flux parallel to the crest (i.e., the normal to

Table S1: Initial conditions and parameters of the bidirectional wind regimes in the numerical simulations: θ and N are the divergence angle and the transport ratio between the two winds. ΔT_N and ΔT_1 are the duration of the dominant and secondary winds.

Sand availability	θ [°]	N	ΔT_1 [t_0]	ΔT_N [t_0]
Erodible sand bed	≤ 90	1	100	100
Erodible sand bed	> 90	1	300	300
Erodible sand bed	≤ 90	1.5	100	150
Erodible sand bed	> 90	1.5	300	450
Erodible sand bed	≤ 90	2	100	200
Erodible sand bed	> 90	2	200	400
Erodible sand bed	≤ 90	5	80	400
Erodible sand bed	> 90	5	90	450
Localized sand source	$0 - 180$	1	300	300
Localized sand source	$0 - 180$	1.5	200	300
Localized sand source	$0 - 180$	2	150	300
Localized sand source	$0 - 180$	2.5	80	200
Localized sand source	$0 - 180$	3	80	240
Localized sand source	$0 - 180$	3.5	80	280
Localized sand source	$0 - 180$	4	80	320
Localized sand source	$0 - 180$	4.5	80	360
Localized sand source	$0 - 180$	5	60	300

crest components cancel each other over the entire duration of the wind reorientation cycle). This finger dune may be classified as a longitudinal dune because it forms an angle of 10.6° with the resultant sand flux on a flat sand bed.

Movie S3. To illustrate dune growth in the fingering mode and the development of superimposed dunes in the bed instability mode, Movie S3 shows the development of bedforms from a local sand source for $\theta = 155°$ and $N = 2$. For this region of the parameter space $\{\theta, N\}$ of bidirectional wind regimes, we observe a set of barchan dunes. If a finger dune starts to extend over short distance away from the localized sand source, it rapidly breaks up into a train of asymmetric barchan dunes (see the steady-state dune fields associated with this initial condition in Figure 2b of the main manuscript).

Movie S4. A surprising observation in the numerical simulation is the formation of longitudinal dunes in the fingering mode for divergence angles $60° < \theta < 90°$. To illustrate this case, Movie S4 shows the development of bedforms from a local sand source for $\theta = 80°$ and $N = 2$ (see the steady-state dune fields associated with this initial condition in Figure 2b of the main manuscript). This dune may be described as a longitudinal dune as it forms an angle of 5.5° with the resultant sand flux on a flat sand bed. The stability and the extension of such a finger-like structure is ensured

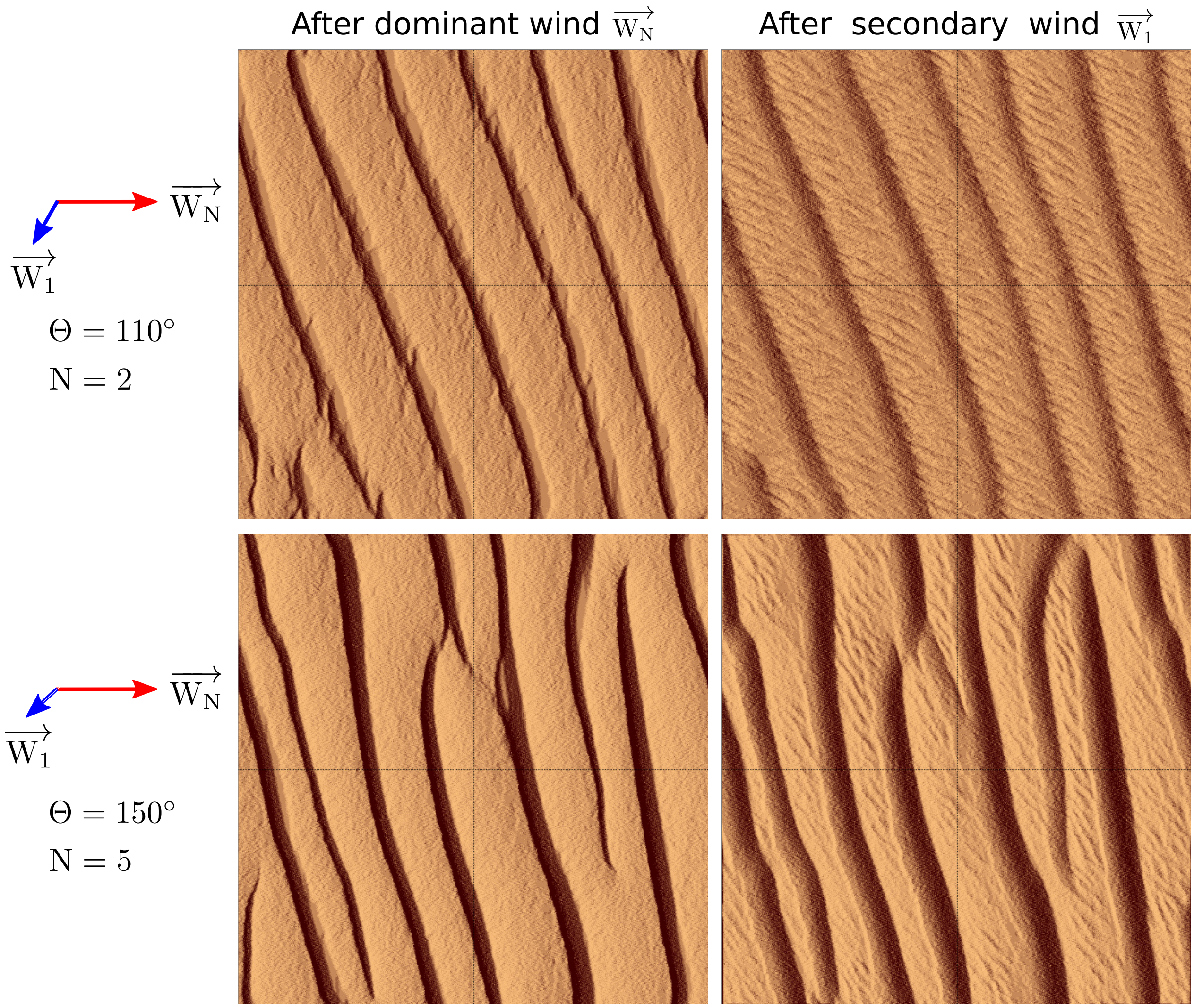

Figure S1: Transient stages of dune fields exposed to bidirectional wind regimes. Comparison of dune fields after the dominant and secondary winds in the numerical model. The red and blue arrows show the sand flux vectors of the dominant and secondary winds, respectively. The cellular space has a square basis of side $L = 600\, l_0$. If the overall dune orientation does not change, note the systematic development of superimposed bedforms perpendicular to the current wind direction. These superimposed bedforms are more visible after the secondary wind because in the bed instability mode the dune orientation is more perpendicular than parallel to the dominant wind. Then, the dune aspect ratio seen by the secondary wind is smaller and superimposed bedforms develop more easily on more gradual slopes.

by winds blowing from both sides of the crest.

3 Supplementary Note 3: Estimation of dune orientation in numerical simulations

As shown in Figure S2 and reported by symbols in Figure 3 of the main manuscript, dune orientation is computed using a 2D spatial autocorrelation of the digital elevation map $H(x, y)$:

$$C(\delta x, \delta y) \sim \sum_{x=0}^{L-1} \sum_{y=0}^{L-1} \left(H(x,y) - \langle H \rangle\right) \left(H(x - \delta x, x - \delta y) - \langle H \rangle\right), \tag{1}$$

where L is the length of the cellular space in horizontal directions expressed in units of l_0. The orientation of dunes is defined as the direction along which the autocorrelation function reaches a maximum (Figure S2). In practice, we calculate the surface integral of the autocorrelation function from the center of the correlogram (i.e., $\delta x = 0$ and $\delta y = 0$) for angles from 0 to 2π using an angular step $\delta\theta = 0.001°$. The selected dune orientation is the one for which the surface integral is maximum. This orientation and the characteristic wavelength of the periodic dune pattern may depend on each other. To check the stability of the predicted dune orientation, we calculate the surface integral for different radius lengths $R/l_0 = \{80, 120, 160\}$ that are several times the characteristic wavelength for the formation of dunes (i.e., $40\,l_0$ as estimated in Narteau et al.[1]).

4 Supplementary Note 4: Effect of diffusion rate on dune orientation in the bed instability mode

The propagation of defects and collisions between adjacent bedforms are known to affect the orientation of linear dune fields[2] and the dynamics of pattern coarsening. To get more stable estimation of dune orientation, we run different simulations for different values of the lateral diffusion rate from $\Lambda_d t_0 = 2 \times 10^{-2}$ to $\Lambda_d t_0 = 1.6$. For each simulation, we compute dune orientation (see Figures S3 and S2) and use the distribution obtained for different Λ_d-values to get the mean orientations and the standard deviations reported in Figure 3A of the main manuscript.

An increasing diffusion rate limits the formation of defects and slows down dune coarsening. Then, as we increase the diffusion rate Λ_d, we can observe a more straight orientation over longer time period (Figure S3). Another solution that has been tested to get more stable orientation is to inject some Gaussian fluctuations of wind orientation around the two principal directions. Thus, a given wind is not only characterized by a mean orientation but also by a standard deviation, which represents the magnitude of its directional variability. Many numerical tests have shown that such a dispersion has a similar effect as that caused by diffusion on the stability of dune orientation[3, 4]. In addition, this wind condition might better account for natural wind regimes. However, this considerably increases the number of rotations in the numerical model and the subsequent numerical artefacts. For this reason, we prefer to play with the diffusion rate as discussed above.

5 Supplementary Note 5: An analytical model for dune orientation

Focusing on dune orientation, Figures 3a and 3b of the main manuscript compare the output of the numerical simulations to the predictions of our analytical model presented in full detail in Courrech du Pont et al.[5]. We just recall here the main characteristics which are particularly relevant to the present study.

We analyze the effect of a bidirectional wind regime on a linear dune of width W and height H (Figure S4). A cartesian system of coordinates $(\vec{i}, \vec{j})$ is defined with respect to the prevailing wind $\overrightarrow{W_N}$ (i.e, $\overrightarrow{W_N} = \|\overrightarrow{W_N}\|\vec{i}$). In this system of reference, the dune crest forms an angle $\alpha \in [0; \pi/2]$

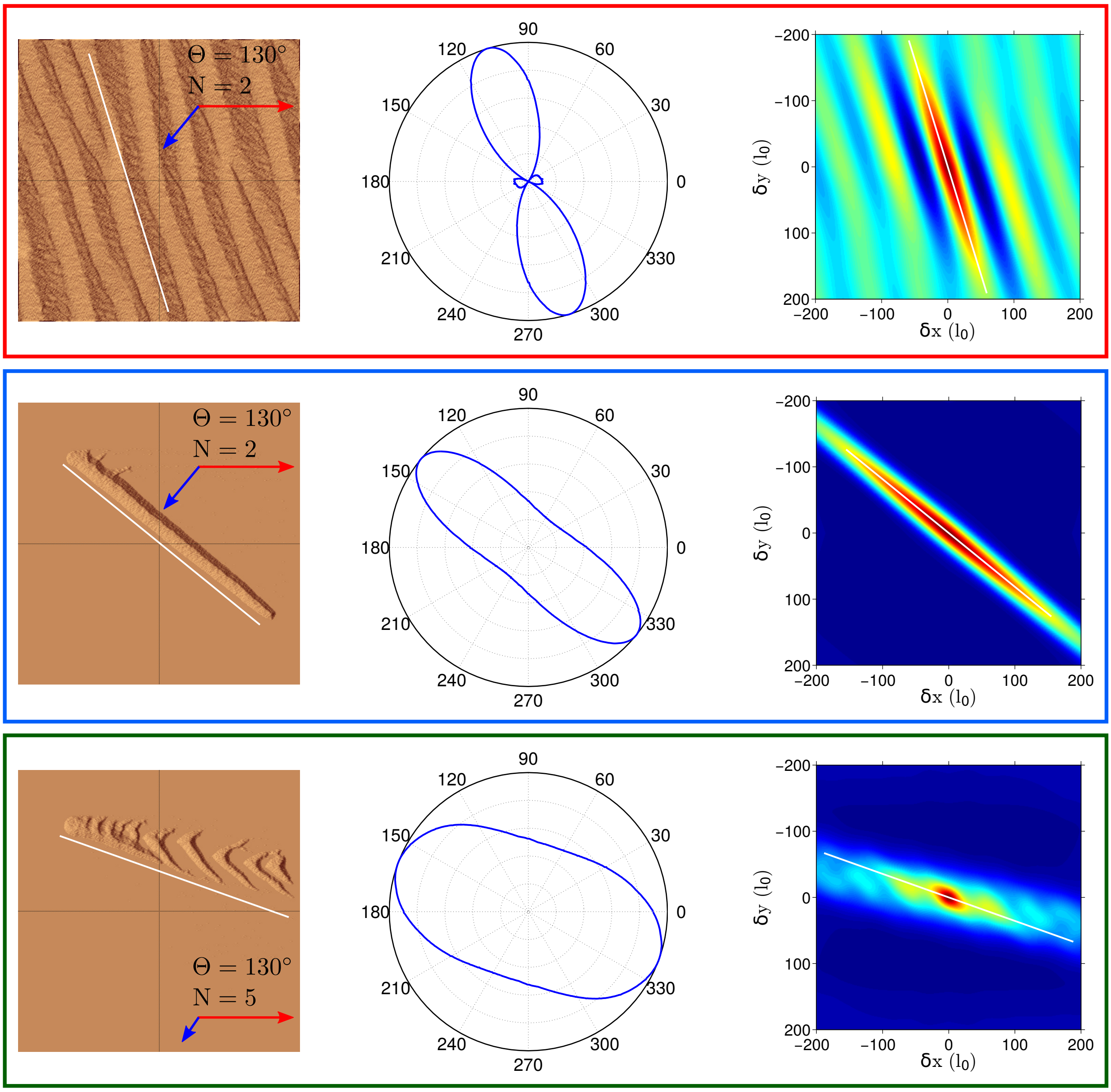

Figure S2: Estimation of dune orientation using 2D spatial autocorrelation. All examples are extracted from the simulations shown in Figures 1 and 2 of the main manuscript. (top) Oblique dune growth from a semi-infinite sediment layer ($\theta = 130°$, $N = 2$). This is a typical measurement of dune orientation in the bed instability mode. (middle) Finger dune growth from a localized sand source ($\theta = 130°$, $N = 2$). This is a typical measurement of dune orientation in the fingering mode. (bottom) Orientation of a train of barchan dunes ($\theta = 130°$, $N = 5$). Note also the asymmetry of the barchans. From top to bottom, each figure shows the dune field topography (left), the 2D spatial autocorrelation function (right) and its angular distribution of energy (center). In all cases, the selected orientation (white lines) is the one along which the autocorrelation function reaches a maximum. Red and blue arrows are the sand flux vectors of primary and secondary winds, respectively.

with the x-axis, the direction of the prevailing wind. As in the numerical model, the two winds $\overrightarrow{W_N}$ and $\overrightarrow{W_1}$ are of equal strength but of different durations $\{\Delta T_{\rm N}, \Delta T_1\}$. Under this condition, the bidirectional wind regime is defined by the divergence angle $\theta \in [0; \pi]$ between the two winds and the transport ratio $N = \Delta T_{\rm N}/\Delta T_1$. The full period of wind reorientation $\Delta T = \Delta T_{\rm N} + \Delta T_1$ is assumed to be small in comparison with the turnover time of the dune H^2/Q_0, where Q_0 is the

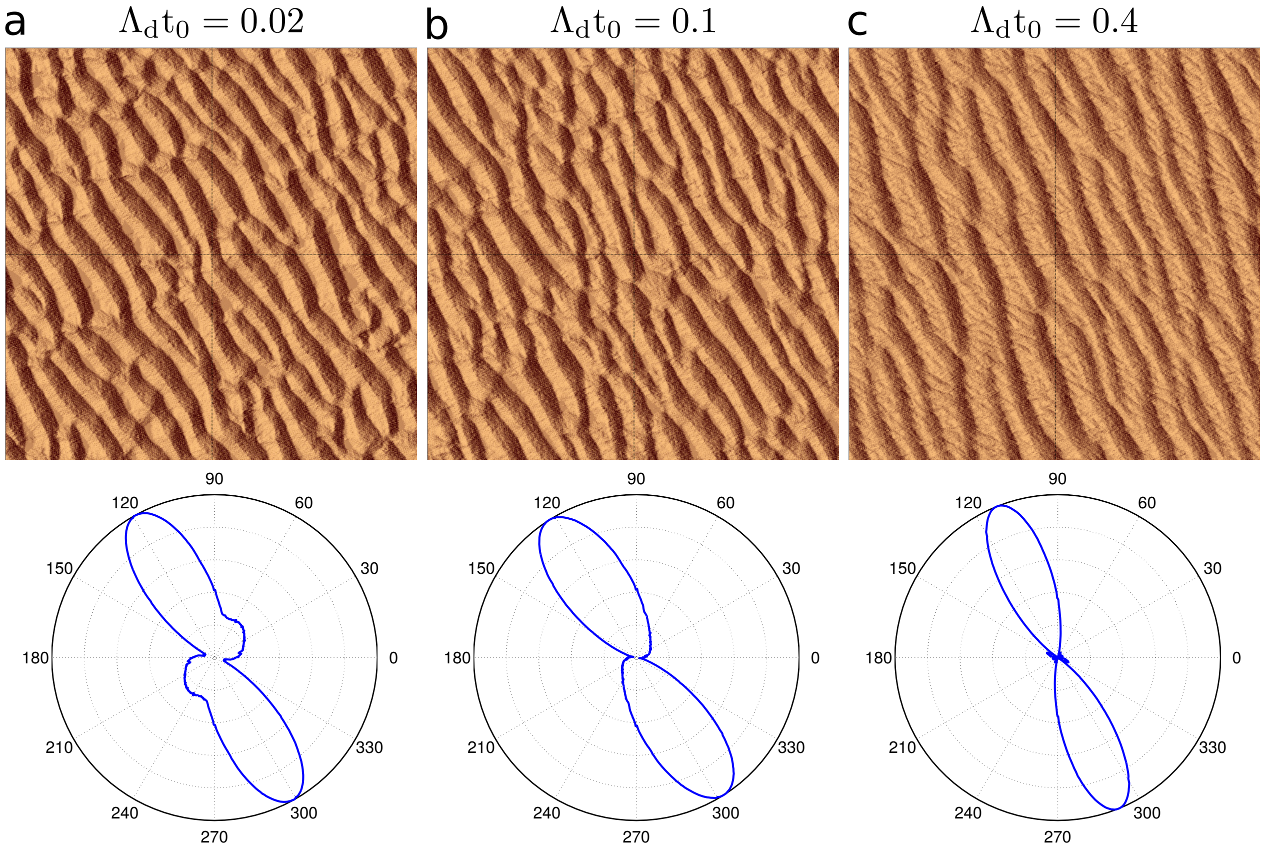

Figure S3: Effect of diffusion rate Λ_d on dune orientation in the bed instability mode for $\theta = 110°$ and $N = 2$. From left to right, each figure shows the dune morphology (top) and the angular distribution of dune orientation (bottom) at $t/t_0 = 4.5 \times 10^3$. The sinuosity of crest lines and the density of defect depend on the value of the lateral diffusion coefficient Λ_d. For smaller diffusion rates, when transverse fluxes are reduced, the bedforms three dimensionality increases because of defect migration and termination creation. Inversely, an increase of lateral fluxes limits height difference between two adjacent layers of cells and tend to form 2D transverse dune fields (i.e., regular crestlines perpendicular to the flow). In practice, we run different simulation for different Λ_d-values to estimate dune orientation and its variability. Note that we could have also consider bimodal wind regime instead of purely bidirectional ones. Fluctuations around the two main wind orientations have the same effect as the diffusion rate. Nevertheless, there are potentially more artefacts related to the rotation of the cellular space in the model.

characteristic sand flux per unit of length over a flat sand bed. Hence, the dune aspect ratio H/W may be considered as a constant during the entire period of wind reorientation, even if the two winds blow alternately from both sides of the dune and periodically modify its summit shape by crest reversals.

A topographic obstacle causes the flow lines to converge on the windward side and to diverge on the lee side. As a result there is a speed-up effect on the top of dunes. Following Jackson and Hunt[6], the fractional speed-up ratio may be approximated by a Taylor expansion in the dune aspect-ratio. In our schematic model described in Figure S4, the two winds do not experience the same dune elevation profile, and therefore, the sand flux at the crest depends on the apparent dune aspect ratio. If the dune height H is the same for both winds, the dune length seen by individual winds may vary. The apparent length of the dune seen by the primary and secondary winds are

$$L_N = \frac{W}{|\sin\alpha|} \qquad \text{and} \qquad L_1 = \frac{W}{|\sin(\theta-\alpha)|}, \tag{2}$$

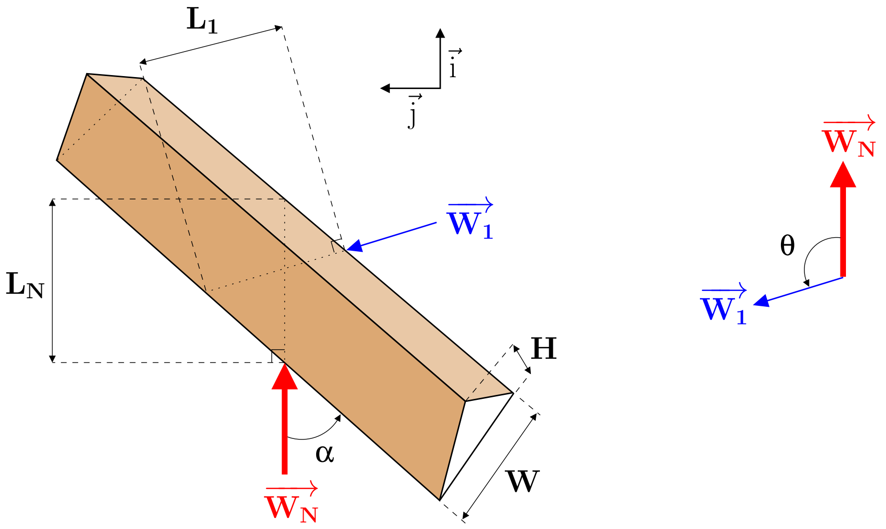

Figure S4: Schematic representation of a linear dune subjected to a bidirectional wind regime. $\overrightarrow{W_N}$ and $\overrightarrow{W_1}$ are the dominant and the subordinate winds, respectively. θ is the angle formed by these two winds. α is the angle between the dominant wind $\overrightarrow{W_N}$ and the linear dune trend. We consider a periodic wind regime with only two changes in flow orientation. Within a full period of wind reorientation, $\Delta T = \Delta T_{\mathrm{N}} + \Delta T_1$, $\overrightarrow{W_N}$ blows N times longer than $\overrightarrow{W_1}$ (i.e., $\Delta T_{\mathrm{N}} = N\Delta T_1$).

respectively. Then, the primary and secondary winds are associated with different sand fluxes at the crest for θ between 0 and 180°. Considering the speed-up effect due to the apparent dune aspect-ratio to the first order and neglecting the transport threshold, the saturated sand flux at the crest produced by the primary wind is

$$\overrightarrow{Q_N} = Q_0 \left(1 + \beta \frac{H}{L_N}\right) \vec{i} = Q_0 \left(1 + \beta \frac{H}{W} |\sin\alpha|\right) \vec{i}, \tag{3}$$

where Q_0 is the value of the saturated sand flux over a flat sand bed and β a dimensionless coefficient that accounts for all the other physical ingredients (e.g., roughness) that affect the speed-up[6, 7]. For the secondary wind, the sand flux at the crest is

$$\overrightarrow{Q_1} = Q_0 \left(1 + \beta \frac{H}{L_1}\right) \left(\cos\theta \vec{i} + \sin\theta \vec{j}\right) = Q_0 \left(1 + \beta \frac{H}{W} |\sin(\theta - \alpha)|\right) \left(\cos\theta \vec{i} + \sin\theta \vec{j}\right). \tag{4}$$

Then, the mean sand flux $\left\langle \overrightarrow{Q_s} \right\rangle$, averaged over a full period of wind reorientation is,

$$\left\langle \overrightarrow{Q_s} \right\rangle = \frac{1}{N+1} \left(N\overrightarrow{Q_N} + \overrightarrow{Q_1}\right). \tag{5}$$

It can be expressed with respect to the dune orientation α as

$$\left\langle \overrightarrow{Q_s} \right\rangle = \frac{Q_0}{N+1} \left(\left(N(1 + \gamma|\sin\alpha|) + \cos\theta(1 + \gamma|\sin(\theta - \alpha)|)\right) \vec{i} + \sin\theta(1 + \gamma|\sin(\theta - \alpha)|)\vec{j} \right), \tag{6}$$

where

$$\gamma = \beta \frac{H}{W}, \tag{7}$$

should be defined as the fractional flux-up ratio because it directly quantifies the increase in sand flux (i.e., $\gamma = (Q - Q_0)/Q_0$). However, we systematically define γ as the "*speed-up*" in what follows to refer to the fractional speed-up ratio proposed by *Jackson and Hunt*[6]. Note that above

the threshold wind speed for motion inception these fractional flux-up and speed-up ratios are directly related to one another according to the transport laws under consideration (see for example Equation 18 and Supplementary Note 7).

Using Equation 6 to estimate the flux at the crest we can now distinguish between the two modes of dune orientation according to local conditions of sediment availability. Overall, these two modes are associated with two distinct dune growth mechanisms: the bed instability and the fingering modes.

Dune growth in the bed instability mode

Let us first consider that there is no limit in sand availability. Linear dunes develop by lateral accretion from the erodible bed of the interdune area. Starting from a flat sand bed, individual winds first produce transverse bedforms that barely interact. After a few cycles of wind directionality, the fields exhibit a prevailing dune trend (see Movie S1 and Figure 2a of the main manuscript). As proposed by *Rubin and Hunter*[8], this prevailing orientation maximizes the overall contribution to dune growth of the different winds.

The dune growth rate may be defined as

$$\sigma = \frac{1}{H} \times \frac{\Delta H}{\Delta t}. \tag{8}$$

Here, we voluntarily use the symbol Δ to indicate that we study changes at the length scale of a dune. This is necessary when considering multidirectional wind regimes and bedforms that need to integrate the entire wind cycle without significant variations in morphology. For the same reason, using the equation of mass conservation $\partial Q/\partial x = -\partial h/\partial t$ at the scale of the dune, one can write

$$\frac{\Delta H}{\Delta t} \propto -\frac{\Delta Q}{\Delta x}. \tag{9}$$

Only a proportionality is expected since the dune also propagates. Thus, we have

$$\sigma \propto -\frac{1}{H} \times \frac{\Delta Q}{\Delta x}. \tag{10}$$

Considering that the sediment flux is negligible at the base of the dune (i.e., $Q_{\text{sat}} \to 0$ in the lee side), it varies from its maximum saturated value Q_{crest} at the crest to 0 over a distance comparable to the downwind length L of the dune (i.e. $\Delta \propto -Q_{\text{crest}}$, $\Delta x = L$). Then, Equation 10 becomes

$$\sigma \propto \frac{Q_{\text{crest}}}{HL}. \tag{11}$$

Such an expression may be generalized to multidirectional wind regimes considering a weighted average of the sediment flux at the crest. Then, for a bidirectional wind regime, the growth rate averaged over the entire period of wind directionality is

$$\begin{aligned} \sigma &\propto \frac{1}{H(N+1)} \left(N \frac{\left\| \overrightarrow{Q_N} \right\|}{L_N} + \frac{\left\| \overrightarrow{Q_1} \right\|}{L_1} \right) \\ &= \frac{Q_0}{(N+1)HW} \left(|\sin(\theta - \alpha)| + \gamma \sin^2(\theta - \alpha) + N|\sin\alpha| + N\gamma \sin^2\alpha \right). \end{aligned} \tag{12}$$

Not surprisingly, the dune growth rate σ is a function of the dune orientation α. In this case, the bed instability mode selects an orientation α_I for which the dune growth rate is maximum, such that

$$\frac{\mathrm{d}\sigma}{\mathrm{d}\alpha} = 0. \tag{13}$$

From Equation 12, we have

$$\frac{d\sigma}{d\alpha} \propto Q_0 \begin{cases} (+\cos(\theta-\alpha) + N\cos(\alpha) - \gamma\sin(2(\theta-\alpha)) + N\gamma\sin(2\alpha)) & \text{for } \theta < 90^\circ, \\ (-\cos(\theta-\alpha) + N\cos(\alpha) - \gamma\sin(2(\theta-\alpha)) + N\gamma\sin(2\alpha)) & \text{for } \theta \geq 90^\circ. \end{cases} \tag{14}$$

Then, in the parameter space $\{\theta,\ N\}$ of bidirectional wind regimes, the dune orientation α_I depends on the γ-value. For example, the solid line in Figure 3a of the main manuscript shows the α_I-values obtained for $\gamma = 1.6$. Using $\gamma = 0$ and $\gamma \to +\infty$, Figure 2 of the supplementary material of Courrech du Pont et al.[5] shows the entire range of possible α_I-values.

Dune growth in the fingering mode

In zones of low sediment availability, the bed instability cannot occur because the dune cannot grow in height from the underlying sediment layer. With a localized sediment supply, dunes may extend from the source due to the sediment flux parallel to the crest. In bidirectional wind regimes, the numerical simulations show the formation of a finger-like structure (see Figure 1b and Figure 2b of the main manuscript as well as Movie S2). These structures elongate in the direction of the mean sand flux at the crest and do not migrate laterally. Hence, using the expression of $\left\langle \overrightarrow{Q_s} \right\rangle$ (Equation 6), the orientation of the dune α_F is given by

$$\tan\alpha_F = \frac{\left\langle \overrightarrow{Q}(\alpha_F) \right\rangle \cdot \vec{j}}{\left\langle \overrightarrow{Q}(\alpha_F) \right\rangle \cdot \vec{i}} = \frac{\sin\theta\,(1+\gamma\sin(\theta-\alpha_F))}{N(1+\gamma\sin\alpha_F) + \cos\theta\,(1+\gamma\sin(\theta-\alpha_F))}. \tag{15}$$

Then, in the parameter space $\{\theta,\ N\}$ of bidirectional wind regimes, the dune orientation α_F depends on the γ-value. For example, the solid line in Figure 3b of the main manuscript shows the α_F-values obtained for $\gamma = 1.6$. Using $\gamma = 0$ and $\gamma \to +\infty$, Figure 4 of the supplementary material of Courrech du Pont et al.[5] shows the entire range of possible α_F-values.

Dune growth rate in the bed instability and the fingering modes

Within the parameter space $\{N,\ \theta\}$ of bidirectional wind regimes, we compute σ_I and σ_F, the growth rate of the bed instability and the fingering modes by injecting $\{\gamma,\ \alpha_I\}$ and $\{\gamma,\ \alpha_F\}$ into Equation 12, respectively. These growth rates are normalized by $Q_0/(HW)$. The dimensionless σ_F/σ_I is used to infer the relative contribution of both dune growth mechanisms to the final morphology of the dune field. By definition, σ_I is always larger than σ_F, so that

$$0 \leq \sigma_F/\sigma_I \leq 1.$$

Figures 4 and 6a in the main manuscript show the σ_F/σ_I-ratio within the parameter space $\{\theta,\ N\}$ of bidirectional wind regimes and around the Tibesti massif (east central Sahara), respectively.

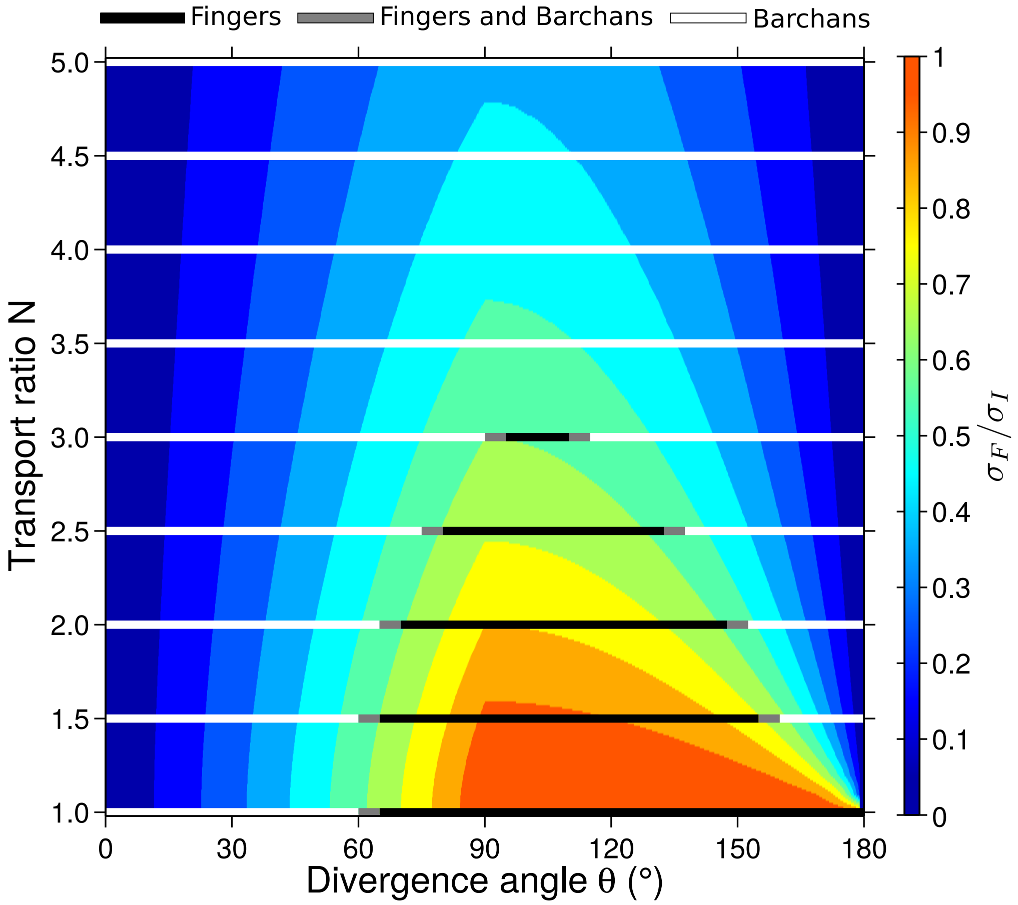

Figure S5: Growth rate ratio σ_F/σ_I in the parameter space $\{\theta,\, N\}$ of bidirectional wind regimes with $\gamma = 0$. The ratio σ_I/σ_F depends on the wind regime only. For conditions of a localized sand source in the numerical model, black and white lines show the zones of the parameter space in which fingers and barchan are observed, respectively. Gray lines indicate transition zones where both dune patterns coexist. This figure can be compared to Figure 4A of the main manuscript.

Figure S5 shows the σ_F/σ_I ratio within the parameter space $\{\theta,\, N\}$ of bidirectional wind regime with $\gamma = 0$. With γ set to zero, σ_F/σ_I is maximum at fixed N when $\theta = 90°$.

Also, we can calculate $\|\overrightarrow{Q_F}\|$, the resultant sediment flux parallel to the crest of finger dunes by injecting $\{N,\, \theta,\, \gamma,\, \alpha_F\}$ into Equation 6, and $\|\overrightarrow{Q_I}\|$, the resultant sediment flux at the crest of dunes in the bed instability mode by injecting $\{N,\, \theta,\, \gamma,\, \alpha_I\}$ into Equation 6. Figure S6 reports how the two major dune types (fingers and trains of barchans) depend on the sediment flux ratio $\|\overrightarrow{Q_F}\|/\|\overrightarrow{Q_I}\|$. It shows a similar behaviour as for the growth rate ratio shown in Figure 4a of the main manuscript.

Let us consider a barchan with two horns in a multidirectional wind regime. Like for a unidirectional wind, these horns point in the mean sand flux direction. It seems reasonable that one horn can elongate in the mean sand flux direction if the horn tip velocity is larger than the velocity of the barchanoid base, i.e., if $\|\overrightarrow{Q_F}\|$ (sand flux for the horn, aligned with the mean sand flux) is larger than $\|\overrightarrow{Q_I}\|$ (sand flux for the base, aligned in the bed instability mode). $\|\overrightarrow{Q_F}\|$ is different from $\|\overrightarrow{Q_I}\|$ because of the speed-up effect. $\|\overrightarrow{Q_F}\| = \|\overrightarrow{Q_I}\| = Q_0$ if $\gamma = 0$. Then, the ratio $\|\overrightarrow{Q_F}\|/\|\overrightarrow{Q_I}\|$ is highly dependent on the σ value and too sensitive to be used as a predicted parameter in the field.

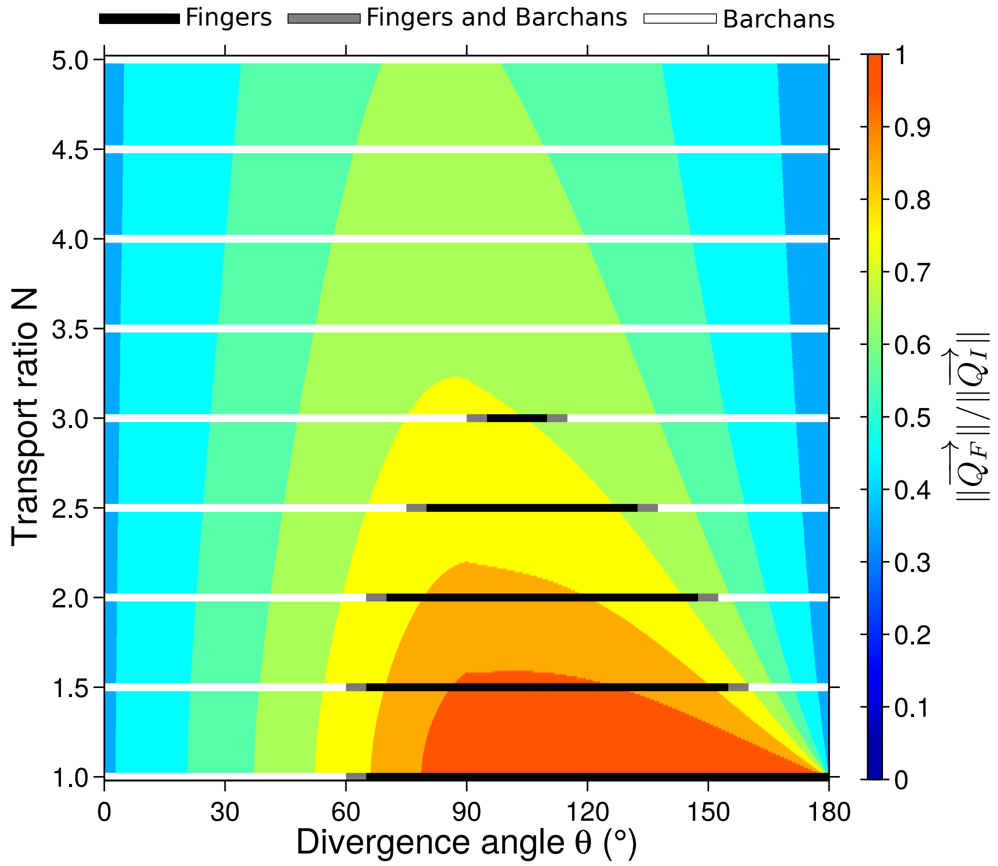

Figure S6: Sediment flux ratio $\|\overrightarrow{Q_F}\|/\|\overrightarrow{Q_I}\|$. The contour plot shows the sediment flux ratio $\|\overrightarrow{Q_F}\|/\|\overrightarrow{Q_I}\|$ between the sediment flux at the crest of dunes growing in the fingering and the bed instability modes using $\gamma = 1.6$. Normalized by Q_0, these dimensionless fluxes are calculated with Equation 6 using the corresponding $\{N,\, \theta,\, \alpha_I,\, \alpha_F\}$-values. The ratio $\|\overrightarrow{Q_F}\|/\|\overrightarrow{Q_I}\|$ depends on the wind regime only. For conditions of a localized sand source in the numerical model, black and white lines show the zones of the parameter space in which fingers and barchan are observed respectively. Gray lines indicate transition ranges.

6 Supplementary Note 6: Predicting sand transport properties and dune orientation from wind data

The wind data around the Tibesti massif used in Figure 6a of the main manuscript are extracted from the ERA-Interim reanalysis, the latest global atmospheric data assimilation model produced by the European Centre for Medium-Range Weather Forecasts (ECMWF). Recent developments of the integrated forecasting system have improved significantly the quality of the outputs of the ERA-Interim project compared to those of the ERA-40 project[9, 10, 11, 12].

The ERA-Interim data are produced for all locations on Earth. This model extends back to 1979 and has been continued to present with a horizontal spatial resolution of 0.25°×0.25° and a time resolution of 6 hours (0: 00, 6: 00, 12: 00, 18: 00 UTC). For this period of time and these resolutions, we only use here the complete time series of 10 m wind data in the terms of the azimuthal and meridional components.

From the wind dataset, we calculate the wind speed u_i and direction $\vec{x}_i$ at different times

$t_1 \leq t_i \leq t_{N_{\mathrm{obs}}}$, $i \in [1; N_{\mathrm{obs}}]$. For each time step i, we calculate the shear velocity

$$u_*^i = \frac{u_i \kappa}{\log(z/z_0)}, \tag{16}$$

where $z = 10\,\mathrm{m}$ is the height at which the wind data as been measured, $z_0 = 10^{-3}\,\mathrm{m}$ the characteristic surface roughness and $\kappa = 0.4$ the von-Kármán constant. The threshold shear velocity value for motion inception can be determined using the formula calibrated by Iversen and Rasmussen[13]

$$u_\mathrm{c} = 0.1\sqrt{\frac{\rho_\mathrm{s}}{\rho_\mathrm{f}} g d}. \tag{17}$$

Using the gravitational acceleration $g = 9.81\ \mathrm{m\,s^{-2}}$, the grain to fluid density ratio $\rho_\mathrm{s}/\rho_\mathrm{f} = 1.97 \times 10^3$ and the grain diameter $d = 180\ \mu\mathrm{m}$, we find $\mathrm{u_c} = 0.19\,\mathrm{m\,s^{-1}}$, which corresponds to a threshold wind speed ten meters above the ground of approximately $4.4\,\mathrm{m\,s^{-1}}$. For each time step i, the saturated sand flux vectors $\overrightarrow{Q_i}$ can be calculated from the relationship proposed by Ungar and Haff[14]

$$Q_\mathrm{sat}(u_*) = \begin{cases} 25\,\dfrac{\rho_\mathrm{f}}{\rho_\mathrm{s}}\sqrt{\dfrac{d}{g}}\left(u_*^2 - u_c^2\right) & \text{for } u_* \geq u_\mathrm{c}, \\ 0 & \text{else.} \end{cases} \tag{18}$$

From the individual saturated sand flux vectors $\overrightarrow{Q_i}$, it is possible to estimate the mean sand flux on a flat erodible bed, also called the drift potential

$$\mathrm{DP} = \frac{\sum_{i=2}^{N_\mathrm{obs}} \left\| \overrightarrow{Q_i} \right\| (t_i - t_{i-1})}{\sum_{i=2}^{N_\mathrm{obs}} (t_i - t_{i-1})}. \tag{19}$$

Averaged over the entire time period, this quantity does not take into account the orientation of the sand fluxes[15]. Then, it is also important to calculate the resultant drift potential,

$$\mathrm{RDP} = \frac{\left\| \sum_{i=2}^{N_\mathrm{obs}} \overrightarrow{Q_i}\, (t_i - t_{i-1}) \right\|}{\sum_{i=2}^{N_\mathrm{obs}} (t_i - t_{i-1})}, \tag{20}$$

which is the norm of the sum of all individual flux vectors. This quantity is strongly dependent on the function of wind directionality.

Using Equations 19 and 20, DP and RDP have units of sand flux. The RDP/DP-value is a non-dimensional parameter which is often used to characterize the directional variability of the wind regimes[16, 17]: RDP/DP $\to 1$ indicates that sediment transport tends to be unidirectional; RDP/DP $\to 0$ indicates that most of the transport components cancel each other. Figure S7 shows the DP, RDP and RDP/DP-values around the Tibesti massif in east central Sahara.

In addition, the computed saturated sand flux vectors $\overrightarrow{Q_i}$ are used to estimate dune orientation using the normal and the parallel to crest components of transport. In what follows, these two components are noted $Q_\perp$ and $Q_\parallel$, respectively.

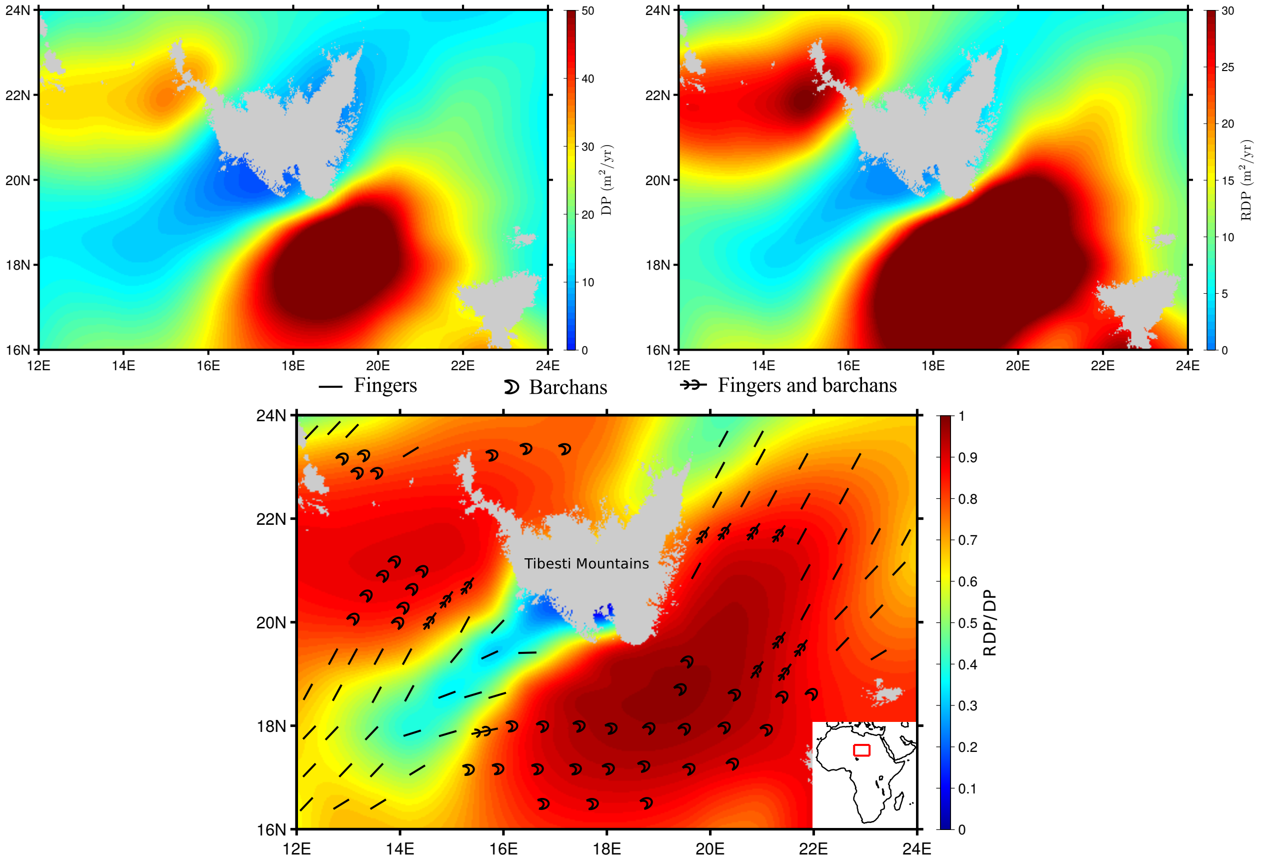

Figure S7: Sand transport properties around the Tibesti massif in east central Sahara: (top left) DP; (top right) RDP; (bottom) RDP/DP (see Equations 19 and 20). Areas higher than 900 m are shown in light gray. The main wind is from the North-East sweeping through western Egypt and central Libya. It is topographically steered by the Tibesti mountains between Libya and Chad and smaller topographic obstacles along the boundaries between Egypt and Libya. Different branches converge downwind, south-west of the Tibesti mountains in the Erg of Fachi Bilma, in Niger. The map used in this figure is generated by GMT (The general mapping tools).

Bed instability mode

To estimate the orientation of the bed instability mode, we estimate the total growth rate $\sigma(\alpha)$ for all possible dune orientations (Equation 12). Considering the angle θ_i of the flux vector $\overrightarrow{Q_i}$, we calculate $Q_\perp(\alpha)$, the total sand flux perpendicular to the crest for all possible crest orientations $\alpha \in [0; \pi]$. Then, we identify the maximum value of $Q_\perp(\alpha)$ that corresponds to the most probable crest orientation α_I of dunes in the bed instability mode. For the bed instability mode, we do not take into account from which side of the dune the wind blows. In fact, all winds contribute to dune growth as soon as they have a normal to crest component of transport. Thus, linear bedforms can develop by lateral accretion and extend longitudinally from both ends as long as there is no limit in sediment availability. Figure S8 shows the predicted bed instability mode around the Tibesti massif (east central Sahara) for $\gamma = 0$ and $\gamma \to +\infty$.

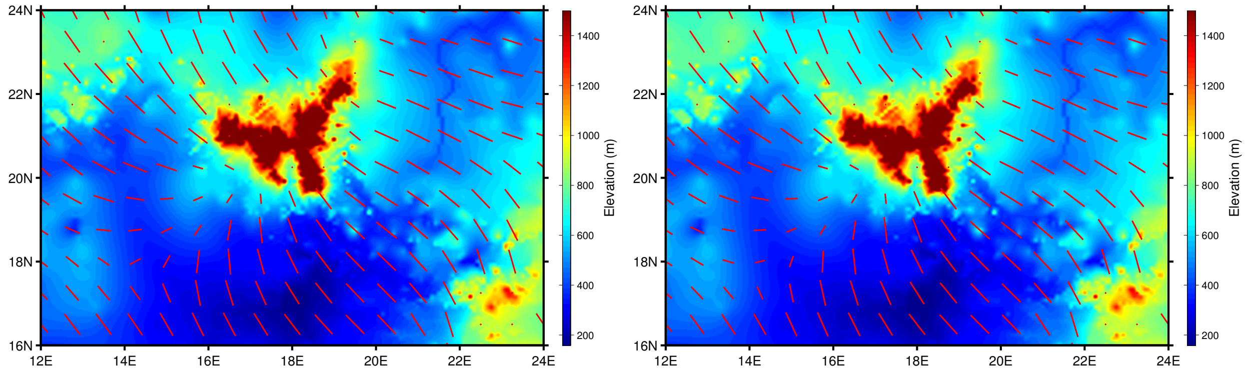

Figure S8: Predicted dune orientation in the bed instability mode around the Tibesti massif in east central Sahara: (left) $\gamma = 0$; (right) $\gamma \to +\infty$. The figure also shows the elevation map but no dune orientation has been calculated for zones with an altitude higher than 900 m.

Fingering mode

The orientation α_F of dunes in the fingering mode is the direction of the mean sand flux $\left\langle \overrightarrow{Q} \right\rangle$ at the crest of the dune (Equation 15). To estimate the orientation of such a fingering mode, we calculate $Q_\perp(\alpha)$ and $Q_\parallel(\alpha)$, the total sand flux perpendicular and parallel to the crest for all possible crest orientations $\alpha \in [0; 2\pi]$. In practice, α_F is the orientation for which the sediment flux perpendicular to the crest vanishes (i.e., $Q_\perp(\alpha) = 0$) and for which the flux parallel to the dune is positive (i.e., $Q_\parallel(\alpha) > 0$). If more than one solution exists, we look for the angle at which the $Q_\parallel$-value is maximum. By definition, when there is no feedback of topography on the flow (i.e., $\gamma = 0$), the orientation of the linear fingering mode α_F is given by the resultant sand transport direction (also called the RDD). There is therefore a range of possible dune orientations from $\gamma = 0$ to $\gamma \to +\infty$. In a vast majority of cases, a γ-value close to 1 gives reasonable estimates of dune orientation[5]. Figure S9 shows the predicted fingering mode around the Tibesti massif (east central Sahara) for $\gamma = 0$ and $\gamma \to +\infty$.

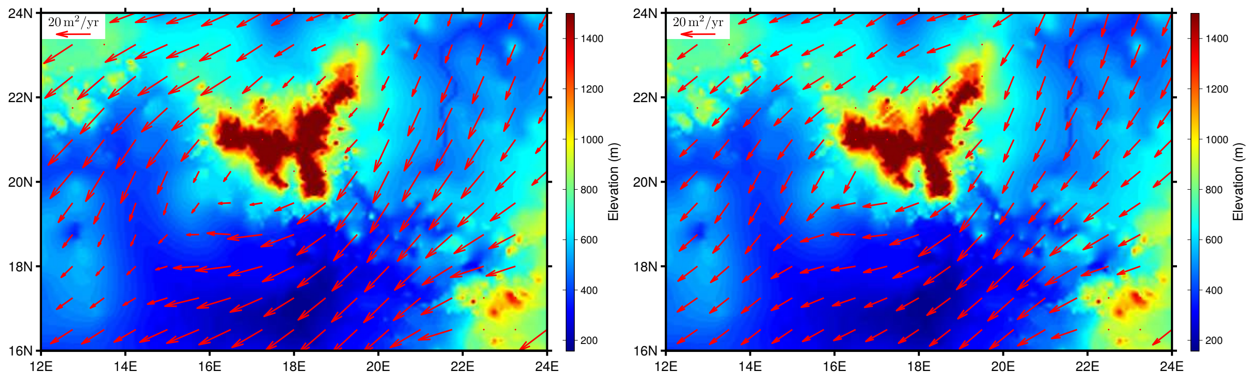

Figure S9: Predicted dune orientation in the fingering mode around the Tibesti massif in east central Sahara: (left) $\gamma = 0$; (right) $\gamma \to +\infty$. The figure also shows the elevation map but no dune orientation has been calculated for zones with an altitude higher than 900 m.

7 Supplementary Note 7: Relationship between the flux-up ratio and the speed-up ratio

For a given wind speed u_0 on a flat sand bed, we can calculate the wind speed u_{crest} at the dune crest using the speed-up ratio $\delta \in [0; 1]$,

$$u_{\text{crest}} = u_0(1 + \delta). \tag{21}$$

By injecting these wind speeds in Equations 16, 17 and 18, we can calculate the sand flux Q_0 and Q_{crest} on a flat sand bed and at the dune crest, respectively. In Supplementary Note 5, we have defined the flux-up ratio as

$$\gamma = \frac{Q_{\text{crest}}}{Q_0} - 1. \tag{22}$$

Hence, we can numerically derive the relationship between the flux-up ratio γ, the speed-up ratio δ and the wind speed u_0 (Figure S10). Because of the transport threshold, γ and δ are proportional only for an asymptotically large wind velocity. The black curve in Figure S10 shows $\gamma = 1.6$.

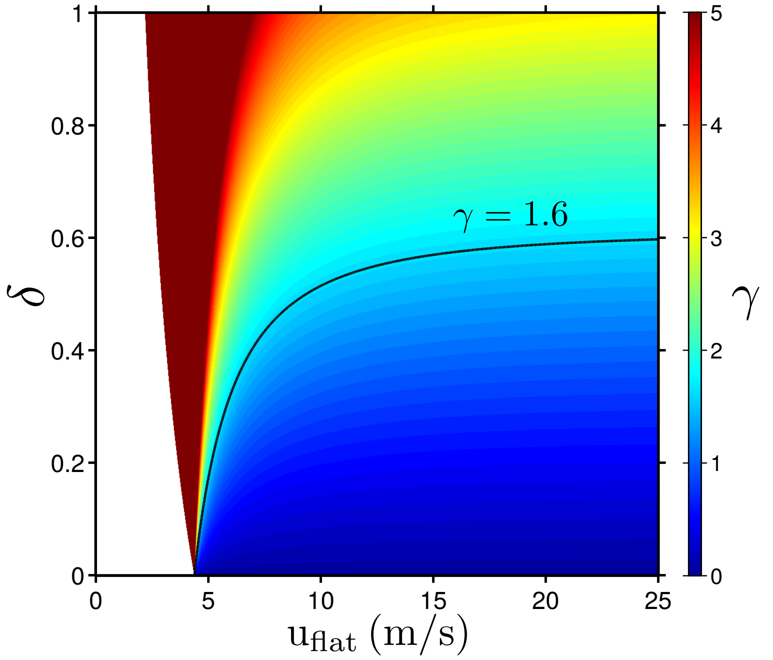

Figure S10: Flux-up ratio γ with respect to the wind speed on a flat sand bed u_0 and the speed-up ratio δ. The black curve is for $\gamma = 1.6$. Note that transport may occur at the dune crest when the wind speed is below the threshold for motion inception on a flat sand bed. This explains the divergence of the γ-value for small wind speed.

References

[1] Narteau, C., Zhang, D., Rozier, O. & Claudin, P. Setting the length and time scales of a cellular automaton dune model from the analysis of superimposed bed forms. *J. Geophys. Res.* **114**, F03006 (2009).

[2] Kocurek, G. & Ewing, R. Aeolian dune field self-organization–implications for the formation of simple versus complex dune-field patterns. *Geomorphology* **72**, 94–105 (2005).

[3] Werner, B. & Kocurek, G. Bed-form dynamics: Does the tail wag the dog? *Geology* **25**, 771–774 (1997).

[4] Zhang, D., Narteau, C. & Rozier, O. Morphodynamics of barchan and transverse dunes using a cellular automaton model. *J. Geophys. Res.* **115**, F03041 (2010).

[5] Courrech du Pont, S., Narteau, C. & Gao, X. Two modes for dune orientation. *Geology* **42**, 743–746 (2014).

[6] Jackson, P. & Hunt, J. Turbulent wind flow over a low hill. *Q. J. Roy. Meteorol. Soc.* **101**, 929–955 (1975).

[7] Britter, R., Hunt, J. & Richards, K. Air flow over a two-dimensional hill: Studies of velocity speed-up, roughness effects and turbulence. *Q. J. Roy. Meteorol. Soc.* **107**, 91–110 (1981).

[8] Rubin, D. & Hunter. Bedform alignment in directionally varying flows. *Science* **237**, 276–278 (1987).

[9] Uppala, S. M. *et al.* The ERA-40 re-analysis. *Q. J. Roy. Meteorol. Soc.* **131**, 2961–3012 (2005).

[10] Simmons, A., Uppala, S., Dee, D. & Kobayashi, S. ERA-Interim: New ECMWF reanalysis products from 1989 onwards. *ECMWF newsletter* **110**, 25–35 (2007).

[11] Berrisford, P. *et al.* The ERA-Interim Archive. *ERA report series* 1–16 (2009).

[12] Dee, D. *et al.* The ERA-Interim reanalysis: Configuration and performance of the data assimilation system. *Q. J. Roy. Meteorol. Soc.* **137**, 553–597 (2011).

[13] Iversen, J. D. & Rasmussen, K. R. The effect of wind speed and bed slope on sand transport. *Sedimentology* **46**, 723–731 (1999).

[14] Ungar, J. & Haff, P. Steady state saltation in air. *Sedimentology* **34**, 289–299 (1987).

[15] Fryberger, S. G. & Dean, G. Dune forms and wind regime. *A study of global sand seas* **1052**, 137–169 (1979).

[16] Pearce, K. I. & Walker, I. J. Frequency and magnitude biases in the 'Fryberger' model, with implications for characterizing geomorphically effective winds. *Geomorphology* **68**, 39–55 (2005).

[17] Tsoar, H. Sand dunes mobility and stability in relation to climate. *Physica A* **357**, 50–56 (2005).
